# Synthesis and Electrochemiluminescence of a Di-Boron Thermally Activated Delayed Fluorescence Emitter

**DOI:** 10.3390/molecules30081718

**Published:** 2025-04-11

**Authors:** Xiaojie Zhou, Jun Cheng, Hongbo Wang

**Affiliations:** 1 Key Laboratory of Flexible Optoelectronic Materials and Technology (Ministry of Education), Center for International Cooperation and Disciplinary Innovation in Sustainable Chemical Engineering, School of Optoelectronic Materials and Technology, Jianghan University, Wuhan 430056, China; 17771987269@163.com; 2 Department of Chemistry, University of Liverpool, Crown Street, Liverpool L69 7ZD, UK

**Keywords:** electrochemiluminescence, thermally activated delayed fluorescence, boron, co-reactant ECL

## Abstract

Recent advances in electrochemiluminescence (ECL) leveraging thermally activated delayed fluorescence (TADF) have highlighted its potential for near-unity exciton harvesting. However, there are still very limited examples of TADF-ECL emitters. We present a rigid diboron-embedded multiple-resonance TADF emitter, which exhibits blue–green emission at 493 nm with a remarkably narrow bandwidth (FWHM = 22 nm) and minimized singlet-triplet energy gap (ΔE_ST_ = 0.2 eV), achieving a 67% photoluminescence quantum yield. DFT calculations confirm the short-range charge transfer, enabling narrowband emission. Co-reactant-dependent ECL shows that tripropylamine (TPrA) improves the ECL efficiency from 11% (annihilation) to 51%, while benzoyl peroxide (BPO) yields 1% due to poor radical stabilization. ECL spectra align with photoluminescence, confirming the singlet-state dominance without exciplex interference. TPrA enhances stable radical formation and energy transfer, whereas BPO induces non-radiative losses. These findings establish molecular rigidity and co-reactant selection as pivotal factors in developing high-performance TADF-ECL systems, providing fundamental guidelines for designing organic electrochemiluminescent materials with optimized exciton harvesting efficiency.

## 1. Introduction

Electrochemiluminescence (ECL) is a process where light is emitted from an electrochemical reaction. Small organic molecules often contain conjugated systems that facilitate electron transfer and light emission, and therefore achieve high ECL efficiency [1]. Organic emitters for ECL have gained significant attention due to their potential applications in biosensing, imaging and light-emitting devices [2,3,4,5,6]. For example, organic emitters have been integrated into ECL biosensors for the detection of biomolecules (e.g., DNA, proteins and enzymes) [7,8]. The high sensitivity and selectivity of ECL make it a powerful tool for bioanalysis. ECL-based imaging techniques using organic emitters have been developed for cellular and tissue imaging, offering high spatial resolution and sensitivity [9,10]. Some organic emitters exhibit enhanced ECL in the aggregated state, leading to the development of AIECL systems with high brightness and stability [11,12,13,14].

Despite significant progress, achieving high ECL efficiency and long-term stability remains challenging, especially for practical applications [15,16]. Scaling up the synthesis of organic emitters and their integration into devices is an area that requires further research. The field of organic emitters for ECL is rapidly advancing, with ongoing research focused on developing new materials, understanding mechanisms and expanding applications. The integration of organic emitters with nanotechnology, biotechnology and device engineering holds great promise for the future of ECL-based technologies.

Thermally activated delayed fluorescence (TADF) materials have emerged as a promising class of emitters for ECL due to their unique ability to harvest both singlet and triplet excitons for light emission [15,17,18]. TADF materials typically exhibit a small energy gap (ΔE_ST_) between the singlet (S_1_) and triplet (T_1_) states, enabling efficient reverse intersystem crossing (RISC) and delayed fluorescence [19,20]. This property makes them highly attractive for ECL applications, where efficient exciton utilization is critical for achieving high luminescence efficiency. In ECL, electrochemically generated radicals (cationic and anionic species) undergo electron transfer reactions to form excited states, which then emit light. For TADF emitters, the excited states can be either singlets or triplets, with the triplets being upconverted to singlets via RISC. TADF materials can utilize both singlet and triplet excitons, leading to higher ECL efficiency compared to traditional fluorescent emitters, which only use singlet excitons [17,21,22,23,24]. TADF emitters have demonstrated significantly higher ECL efficiencies compared to conventional fluorophores due to their ability to harvest triplet excitons. This is particularly important in ECL, where triplet states are often generated in large quantities. TADF materials with a small ΔE_ST_ facilitate efficient RISC, leading to strong delayed fluorescence and improved ECL performance. Recent studies have demonstrated that TADF emitters can achieve superior ECL efficiencies compared to traditional ruthenium complexes (e.g., Ru(bpy)_3_^2+^), which are widely used in ECL [17,23,25]. To minimize ΔE_ST_, TADF emitters are specifically designed with spatially separated donor and acceptor moieties. Carbazole derivatives, for instance, serve as highly effective donor units in TADF-ECL systems owing to their robust electron-donating capacity and chemical stability [26], while triazine derivatives are frequently employed as acceptor components due to their strong electron-withdrawing characteristics [27]. Notably, metal-free TADF materials are particularly attractive for ECL due to their low cost and environmental friendliness. A unique advantage of certain TADF systems lies in their aggregation-induced ECL behavior, where the emission intensity amplifies in the aggregated state—a property that renders them particularly suitable for solid-state ECL devices [11,14,16,28]. These advancements position TADF materials as a transformative innovation in ECL technology, offering high efficiency, tunable emission profiles and the capacity to utilize triplet excitons that are typically lost in conventional systems. However, challenges persist, as TADF materials may undergo electrochemical degradation under operational conditions, posing limitations for their long-term stability in practical ECL applications.

While challenges remain, ongoing research is rapidly addressing these issues, paving the way for the widespread adoption of TADF-ECL in biosensing, imaging and other applications.

Herein, we design and synthesize a rigid diboron-embedded multiple-resonance TADF emitter. Photophysical properties are investigated and DFT calculations are also carried out to figure out the electronic structure and energy gap. Subsequent investigations systematically evaluate annihilation-driven and co-reactant-assisted ECL processes, encompassing quantitative assessments of ECL efficiency, potentials and, most importantly, their ECL spectra. Oxidizing and reducing co-reactants (tripropylamine and benzoyl peroxide) are comparatively investigated to elucidate their distinct roles in stabilizing radical intermediates and mediating energy transfer pathways. ECL spectra are studied to further interrogate the electrochemical reaction process by correlating emission profiles with excited-state dynamics. These mechanistic insights advance the molecular-level comprehension of TADF-ECL phenomena and establish actionable guidelines for engineering high-performance narrowband organic emitters, thereby accelerating their implementation in precision biosensing and energy-efficient optoelectronic systems.

## 2. Results

### 2.1. Design and Synthesis of a Diboron-Embedded Emitter

Rigid structures are often employed to reduce non-radiative decay and enhance photoluminescence quantum yields (PLQYs), which directly impact ECL efficiency. The previously reported rigid structure (DtBuCzB) as a control multiple-resonance thermally activated delayed fluorescence (MR-TADF) material was selected for high luminescence efficiency [26,27]. At the same time, the MOPV dye was chosen to bridge two MR-TADF units due to its rigid structure, which effectively reduced the non-radiative process [14,28]. Furthermore, the long alkyl chains incorporated into MOPV were designed to improve the solubility of BN-MOPV and reduce the π–π stacking of molecules to avoid aggregation-caused quenching of BN-MOPV at a high concentration.

The diboron-embedded emitter BN-MOPV was synthesized through a conventional Suzuki–Miyaura coupling reaction using DtCzB-Bpin and BMOPV as starting materials (shown as Figure 1). DtCzB-Bpin and BMOPV were synthesized according to the reported process [26,27,29]. All those intermediates and the product were characterized by ^1^H and ^13^C NMR spectra, as well as by high-resolution mass spectroscopy (shown in Supplementary Figures S1–S14).

### 2.2. Photophysical Properties

**Figure 2:**
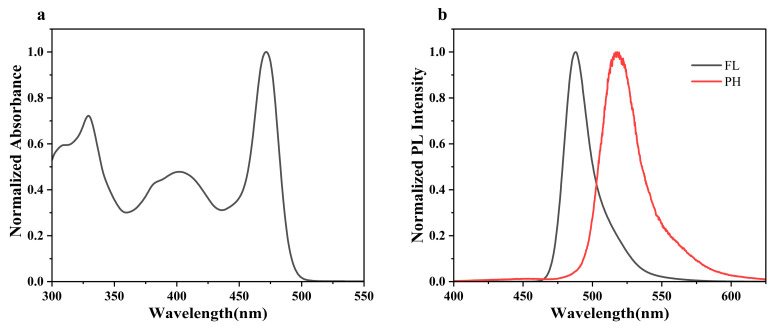
(**a**) Ultraviolet–visible (UV–vis) absorption, (**b**) fluorescence (FL) spectra and phosphorescence (PH) spectra of BN-MOPV measured in toluene solutions (10^−5^ M).

The photophysical properties of BN-MOPV were investigated by UV–visible absorption, fluorescence and phosphorescence spectra (Figure 2), and the results are summarized in Table 1. BN-MOPV exhibits three distinct absorption bands spanning 300–350 nm, 360–430 nm and 450–500 nm in dilute toluene solution (Figure 2a). The first two spectral features (300–350 nm and 360–430 nm) originate primarily from the MOPV bridge architecture, aligning closely with the reported absorption characteristics of MOPV derivatives [29]. In contrast, the narrow peak observed at 450–500 nm arises from charge transfer transitions within the DtBuCzB framework.

The fluorescence and phosphorescence spectra of BN-MOPV reveal their maximum emissions, centered at 493 nm and 517 nm, respectively. The fluorescent component demonstrates a high absolute photoluminescence quantum yield (Φ_PL_) of 67% under air (88% under argon), indicative of efficient radiative recombination and suppressed non-radiative decay pathways. Notably, both emissions exhibit narrowband profiles, as evidenced by full-width-at-half-maximum (FWHM) values of 22 nm (fluorescence) and 32 nm (phosphorescence), suggesting suppressed structural relaxation and enhanced rigidity in the excited states. The optical bandgap, calculated from the spectral onset of these emissions, was determined to be 2.77 eV, aligning with the energy range typical for organic optoelectronic materials. Crucially, the energy splitting (ΔE_ST_) between singlet (S_1_) and triplet (T_1_) excited states was resolved as 0.20 eV, a critical parameter for TADF behavior. This modest ΔE_ST_ value facilitates efficient reverse intersystem crossing (RISC) processes, positioning BN-MOPV as a promising candidate for highly efficient emitters. The observed spectral narrowing in fluorescence further implies reduced conformational disorder in the S_1_ state, potentially arising from the constrained geometry of the MOPV-bridged DtBuCzB framework. These findings corroborate the design strategy of integrating multi-resonant donor–acceptor units to achieve simultaneous narrowband emission and small singlet-triplet energy gaps. Additionally, the emission spectra of BN-MOPV were recorded under distinct atmospheric conditions (air vs. argon), as illustrated in Figure S15. The results reveal a markedly stronger emission intensity in an argon environment compared to air. This pronounced difference stems from oxygen-mediated quenching of triplet excitons—a phenomenon that further supports the presence of TADF characteristics in BN-MOPV.

### 2.3. Theoretical Calculation

**Figure 3:**
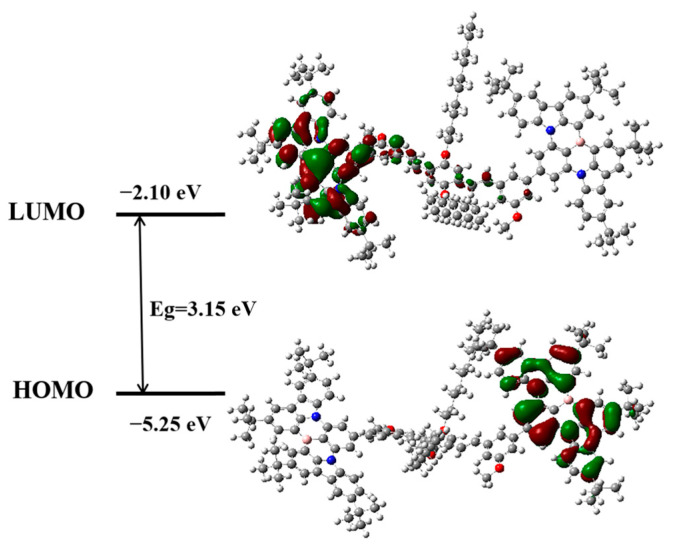
HOMO and LUMO orbital distributions of product BN-MOPV.

To elucidate the electronic configuration and similar multi-resonant emission characteristics of BN-MOPV, density functional theory (DFT) simulations were executed at the B3LYP/6-31+G(d) level using Gaussian 09. The calculated frontier molecular orbital (FMO) yielded a LUMO energy of −2.10 eV, localized predominantly on one boron–nitrogen (BN) unit, and a HOMO energy of −5.25 eV, distributed across the opposing BN moiety, resulting in a theoretical bandgap (*E*_g_) of 3.15 eV (Figure 3). Notably, both HOMO and LUMO electron density maps exhibit resonance patterns analogous to the monomeric DtBuCzB structure, with characteristic delocalization nodes centered on boron atoms [30,31]. This orbital segregation resembles multi-resonance (MR) TADF-like behavior but is mechanistically distinct from genuine MR-TADF systems.

Comparative analysis with electrochemically derived energies (HOMO = −2.50 eV, LUMO = −5.20 eV, *E*g = 2.70 eV) reveals a systematic underestimation of the computational bandgap, showing some difference with known limitations of B3LYP in describing charge-transfer excited states (shown in Supplementary Table S1). The conserved spatial distribution of FMOs across both computational and experimental frameworks validates the similar intrinsic multiple-resonance nature of BN-MOPV. Critically, the resonant electron density modulation around boron centers—mirroring the monomeric DtBuCzB system—confirms the preservation of MR-TADF attributes despite structural extension via MOPV bridging.

The synergy between localized FMO distribution and global π-conjugation creates a unique electronic landscape: (i) HOMO-LUMO spatial separation minimizes orbital overlap, reducing radiative recombination barriers; (ii) boron-centered resonance enhances spin-orbit coupling while maintaining small singlet-triplet energy splitting (Δ*E*_ST_ ≤ 0.2 eV). These computational insights rationalize the compound’s dual capability for narrowband emission and efficient up-conversion from triplet states, positioning BN-MOPV as a prototypical design for heavy atom-free TADF systems.

### 2.4. Electrochemistry and Annihilation ECL

**Figure 4:**
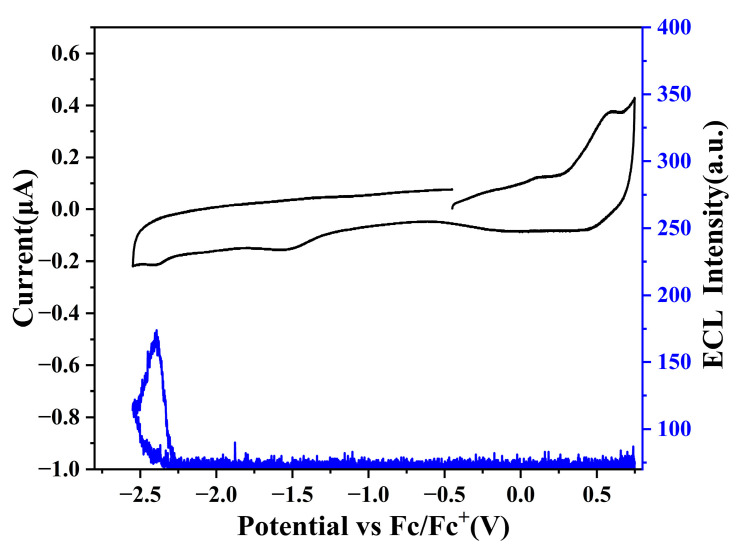
CV and ECL-voltage curve of 0.05 mM BN-MOPV in acetonitrile and benzene (1:1) with 0.1 M tetrabutylammonium hexafluorphosphate (TBAP) as the supporting electrolyte recorded at a scan rate of 0.1 V/s positively.

The electrochemical profile of BN-MOPV was investigated via cyclic voltammetry (CV) to identify its redox characteristics relevant to ECL. During anodic scanning (0.75 to −2.55 V), a single irreversible reduction process was observed at −2.30 V, while cathodic polarization (−2.55 to 0.75 V) revealed an irreversible oxidation event centered at 0.30 V (Figure 4). Notably, a reduction-like feature at −1.52 V was attributed to solvent/electrolyte interactions, as confirmed by control experiments exhibiting identical voltammetric behavior in the absence of BN-MOPV (shown in Supplementary Figure S16). This observation underscores the necessity of distinguishing intrinsic molecular redox activity from background electrolyte processes.

The electrochemical window of BN-MOPV is thus defined by two irreversible redox couples: an oxidation peak at 0.30 V and a reduction peak at −2.30 V, with no additional redox events attributable to the compound itself. The irreversibility of these processes suggests kinetic limitations in charge transfer or structural reorganization during redox cycling, a common feature in multi-resonant systems with rigid π-conjugated frameworks. These results provide critical parameters for ECL studies, where the oxidation-reduction potential gap (~2.6 V) may enable insufficient energy for excited-state generation.

The electrochemical and ECL behaviors of BN-MOPV were systematically probed using CV and simultaneous ECL detection, as illustrated in Figure 4. Cyclic voltammograms acquired in a 1:1 acetonitrile/benzene electrolyte (0.1 M TBAP) reveal irreversible oxidation and reduction peaks for all tested compounds, a characteristic attributed to kinetic limitations in charge transfer or structural reorganization during redox cycling. Notably, this electrochemical irreversibility correlates with diminished ECL activity, as evidenced by the low ECL intensity (<170 counts) observed for BN-MOPV and a relative ECL quantum yield of 11% compared to the Ru(bpy)_3_(PF_6_)_2_ reference system. Thermodynamic analysis revealed a critical discrepancy: the annihilation energy (ΔH_ann_ = E_Ox_ − E_Red_ − 0.16 = 2.44 eV) fell short of the singlet excited-state energy (E_S_ = 2.58 eV), yielding a negative energy margin (ΔH_ann_ − E_S_ = −0.14 eV). This thermodynamic insufficiency directly explains the attenuated ECL output, as the annihilation process lacks the requisite energy to populate the emissive singlet state efficiently.

The rigid molecular architecture, while beneficial for narrowband emission, imposes kinetic barriers to redox reversibility. Radical cations and anions generated during anodic and cathodic scans exhibit limited stability, leading to rapid recombination or parasitic side reactions before annihilation.

### 2.5. ECL of Co-Reactant System

In the realm of ECL, the integration of co-reactants such as benzoyl peroxide (BPO) or tri-n-propylamine (TPrA) has emerged as a pivotal strategy to amplify the ECL efficiency of organic compounds. These co-reactants play a dual role by not only participating in the electrochemical redox reactions but also facilitating the generation of a plethora of excited states, which are quintessential for the emission process.

**Figure 5:**
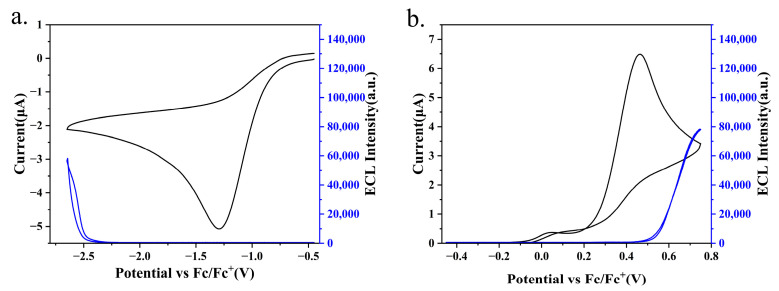
CVs and the corresponding ECL-voltage curves for 0.05 mM products BN-MOPV in the presence of (**a**) 5 mM BPO and (**b**) 5 mM TPrA in 1:1 acetonitrile/benzene with 0.1 M TBAP as the supporting electrolyte at a scan rate of 0.1 V/s.

The integration of BPO as a co-reactant significantly enhanced the ECL performance of the BN-MOPV system. CV analysis (Figure 5a) revealed a distinct reduction peak at −1.51 V, attributable to the electrochemical reduction in BPO. Notably, the reduction peak of BN-MOPV at −2.59 V was obscured due to the substantial excess of BPO (5 mM, ~100-fold higher concentration than BN-MOPV), which dominated the electrochemical processes in the cathodic potential window.

The ECL intensity of BN-MOPV with BPO reached 58,100 a.u., a remarkable enhancement (342-fold) compared to the annihilation-driven ECL pathway (170 a.u.). This amplification arises from the co-reactant mechanism, wherein BPO participates in sequential redox reactions to generate reactive intermediates (e.g., BPO-derived radicals) that react with BN-MOPV radicals. By circumventing the need to scan the full redox potential window (as required in annihilation pathways), the co-reactant strategy minimizes the time interval between radical interactions, thereby reducing radical decay and parasitic side reactions. This kinetic advantage promotes efficient formation of the excited-state species, leading to higher ECL intensities.

**Figure 6:**
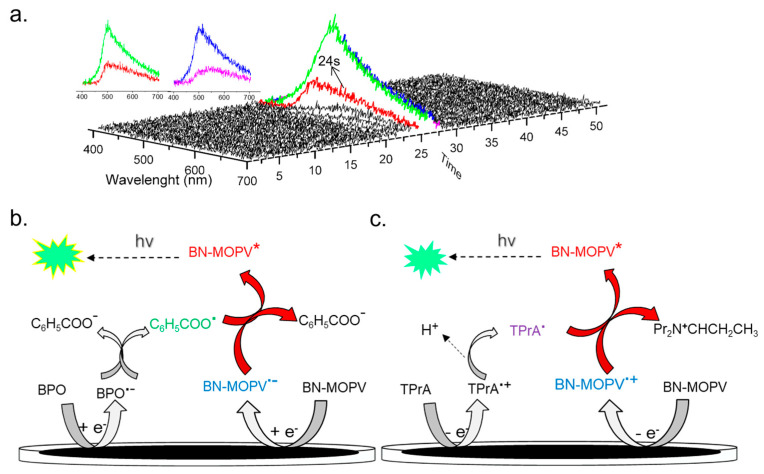
(**a**) Spooling ECL spectra of the BN-MOPV/BPO system (0.05 mM BN-MOPV and 5 mM BPO) in acetonitrile and benzene (1:1) with 0.1 M TBAP as the supporting electrolyte at 0.1 V/s with one spectrum taken every 1 s, where the inset shows an overlay of individual spectra all centering at one wavelength λ_max_ = 500 nm. (**b**) The proposed mechanisms of the BN-MOPV/BPO system and (**c**) BN-MOPV/TPrA system.

Despite the substantial ECL intensity gain, the relative ECL quantum yield (Φ_ECL_) remained modest (~1%), suggesting limitations in the energy transfer or radiative decay efficiency of the excited state. Nevertheless, the intensity enhancement enabled the acquisition of spooling ECL spectra (Figure 6a), which were undetectable in annihilation-driven systems. The spectra exhibited a dominant emission peak at 500 nm, closely aligned with the PL peak of BN-MOPV (493 nm). This congruence confirms that the monomeric excited state (BN-MOPV*) is the sole emissive species generated during the ECL process, with no evidence of excimer or exciplex formation.

The ECL mechanism of the BN-MOPV/BPO system involves sequential redox steps mediated by the co-reactant. As shown in Figure 6b, BPO is first reduced at −1.4 V, forming a benzoate radical anion (C_6_H_5_CO_2_^•−^), which undergoes rapid O–O bond cleavage to generate a strongly oxidizing benzoate radical (C_6_H_5_CO_2_^•^) and a benzoate anion. Upon further cathodic scanning to −2.55 V, BN-MOPV is reduced to its radical anion (BN-MOPV^•−^). BN-MOPV^•−^ reacts with the pre-formed C_6_H_5_CO_2_^•^ via radical annihilation, producing the excited-state BN-MOPV* (BN-MOPV^•−^ + C_6_H_5_CO_2_^•^ → BN-MOPV* + C_6_H_5_CO_2_^−^). Radiative decay of BN-MOPV* to the ground state results in light emission at 500 nm, consistent with its PL profile. Spooling ECL spectroscopy confirms the monomeric excited state as the sole emissive species, with no excimer/exciplex contributions. The co-reactant strategy enhances ECL by synchronizing radical generation, minimizing side reactions and bypassing the need for full redox cycling of BN-MOPV.

The integration of TPrA as a reducing co-reactant markedly enhanced the ECL performance of BN-MOPV, demonstrating the critical role of co-reactant chemistry in optimizing exciton generation. Cyclic voltammetry profiles of the BN-MOPV/TPrA system (5 mM TPrA, Figure 5b) exhibit a dominant oxidation peak at 0.47 V (onset: 0.25 V), attributable to TPrA oxidation, which obscures the intrinsic oxidation of BN-MOPV (0.30 V) due to the 100-fold excess of the co-reactant. This selective electrochemical activation confines the reaction pathway to TPrA-driven radical generation, thereby circumventing stability issues associated with BN-MOPV’s irreversible redox processes.

The co-reactant-assisted ECL system exhibited a dramatic intensity increase to 78,400 a.u.—representing a 461-fold amplification compared to the annihilation pathway (170 a.u.). Concurrently, the relative quantum yield improved significantly to 51%, far exceeding the 1% efficiency observed in the BPO-based system. This marked enhancement highlights TPrA’s superior ability to stabilize reactive intermediates and drive efficient radical annihilation processes [32]. Mechanistically, TPrA undergoes oxidation to form a cation radical (TPrA^•+^), which rapidly deprotonates to yield a strong reducing agent (TPrA^•^). Simultaneously, BN-MOPV is oxidized to its radical cation (BN-MOPV^•+^). The TPrA^•^ species reduces BN-MOPV^•+^, populating the singlet excited state (BN-MOPV*). Radiative decay of BN-MOPV* to the ground state produces the observed ECL emission (Figure 6c).

The stark efficiency contrast between TPrA (51%) and BPO (1%) systems highlights the critical dependence of ECL performance on co-reactant redox dynamics and intermediate stability. These findings position TPrA as an optimal co-reactant for TADF emitters, offering a blueprint for leveraging sacrificial reductants to amplify ECL in kinetically constrained organic systems.

## 3. Materials and Methods

### 3.1. Synthesis of a Diboron-Embedded Emitter

Intermediates DtCzB-Bpin, DOP-BMP and BMOPV were synthesized according to the reported procedure [26,27,29]. Detailed synthetic protocols and characterization data can be found in the Supplementary support information. ^1^H (400 MHz) and ^13^C (100 MHz) NMR spectra were recorded with an AscendTM 400 MHz spectrometer (Bruker). ^1^H and ^13^C NMR spectra were referenced relative to tetramethyl-silane (TMS) using the residual non-deuterated NMR solvent signal. For further analysis, the Microflex LRF mass spectrometer (Bruker, Billerica, MA, USA), a high-performance MALDI-TOF system, was employed to determine the molecular weights and assess the purity of the synthesized compounds. This instrument provides precise and reliable data, complementing the NMR spectroscopy results for comprehensive structural characterization.

Synthesis of compound BMOPV

In a 100 mL standard-mouth round-bottomed flask with a magnetic stirrer, transfer DOP-BMP (2.15 g, 8.2 mmol, 2.8 eq), 3-bromo-5-methoxybenzaldehyde (623 mg, 2.896 mmol, 1 eq) and potassium tert-butoxide (650 mg, 5.792 mmol, 2 eq) under dry argon. Add 30 mL of deoxygenated THF via a constant-pressure funnel. Stir at room temperature for 6 h. After washing with water several times, concentrate the mixture under vacuum. Purify the crude product by silica gel chromatography with petroleum ether/dichloromethane (4:1, *v/v*). Collect the product fractions, concentrate and dry under vacuum for 12 h to obtain yellow crystalline (398 mg, 34% yield). ^1^H NMR (400 MHz, Chloroform-d) δ 7.44 (s, 1H), 7.40 (s, 1H), 7.27 (s, 2H), 7.07 (s, 2H), 7.05 (s, 1H), 7.01 (s, 1H), 6.96 (d, *J* = 8.8 Hz, 4H), 4.04 (t, *J* = 6.6 Hz, 4H), 3.83 (s, 6H), 1.86 (q, *J* = 7.1 Hz, 4H), 1.55 (s, 4H), 1.37–1.18 (m, 24H), 0.90–0.85 (m, 6H).^13^C NMR (100 MHz, Chloroform-d) δ 160.48, 151.18, 140.84, 127.56, 126.65, 125.19, 123.07, 122.05, 115.89, 111.17, 110.86, 69.53, 55.50, 31.90, 29.65 (d, *J* = 8.4 Hz), 29.48 (d, *J* = 2.6 Hz), 29.35, 26.33, 22.70, 14.12.

Synthesis of compound BN-MOPV

In a 100 mL three-necked flask with a magnetic stirrer and spherical condenser, dissolve BMOPV (500 mg, 0.617 mmol, 1 eq) in 30 mL of THF. Add DtCzB-Bpin (992 mg, 1.296 mmol, 2.1 eq), Na_2_CO_3_ (654 mg, 6.171 mmol, 10 eq) and tetrakis(triphenylphosphine)palladium (72 mg, 0.0617 mmol, 0.1 eq). Transfer the mixture to the flask, stir under nitrogen while maintaining an argon atmosphere and reflux at 85 °C for 12 h. After cooling to room temperature, filter off insoluble inorganic salts, extract the reaction mixture with dichloromethane and concentrate the organic phase under reduced pressure. Purify the crude product via silica gel chromatography using a petroleum ether/dichloromethane elution system (3:1, *v/v*) to obtain the final product as a yellow powder (238 mg, 20% yield). ^1^H NMR (400 MHz, Chloroform-d) δ 9.16 (s, 4H), 8.56 (s, 4H), 8.48 (d, *J* = 8.6 Hz, 8H), 8.29 (s, 4H), 7.71–7.64 (m, 8H), 7.38 (s, 3H), 7.33 (s, 1H), 7.28 (s, 4H), 4.15–4.10 (m, 4H), 4.05 (s, 6H), 1.86 (t, *J* = 7.6 Hz, 4H), 1.68 (d, *J* = 2.3 Hz, 36H), 1.53 (d, *J* = 2.1 Hz, 36H), 1.26 (s, 14H), 1.05 (s, 14H), 0.74 (t, *J* = 6.9 Hz, 6H).^13^C NMR (101 MHz, Chloroform-d) δ 160.50, 151.33, 146.27, 145.37, 144.68 (d, *J* = 4.9 Hz), 143.92, 141.78, 140.22, 138.35, 129.83, 128.79, 127.17, 126.96, 124.55, 123.65, 121.72, 120.65, 119.48, 117.31, 114.19, 113.34, 110.94, 110.63, 107.31, 69.70, 55.60, 35.19, 34.81, 32.20, 31.82 (d, *J* = 3.7 Hz), 29.63, 29.48 (d, *J* = 8.3 Hz), 29.26, 26.40, 22.59. MS (MALDI−TOF): calcd. For C_136_H_156_B_2_N_4_O_4_ *m/z* = 1931.2313 [M]+, found: *m/z* =1931.6032 [M]+.

### 3.2. Determination of Photophysical Properties

All experimental evaluations were carried out in toluene solvent systems (1 × 10^−5^ M concentration). Optical absorption profiles were acquired with a UV-1500 spectrophotometric system (Shanghai Macy Instrument Co., Ltd., Shanghai, China). Steady-state photoluminescence and phosphorescence emission spectra were recorded in 77K and excited by 331 nm using an Edinburgh FLS5 fluorescence spectrometer (Techcomp Instrument Co., Ltd., Edinburgh, UK). Absolute photoluminescence quantum yields (PLQYs) under atmospheric conditions were determined via the spectrometer’s integrated sphere module.

### 3.3. DFT Calculations

Theoretical calculations were performed to elucidate the electronic structure and molecular geometry of BN-MOPV. Ground-state geometric optimization was conducted using density functional theory (DFT) implemented in Gaussian 16, Revision C.01, employing the B3LYP hybrid functional paired with the 6-31G(d) basis set. This combination balances computational accuracy and resource efficiency for medium-sized organic systems. All calculations were initiated from pre-optimized molecular structures, with convergence criteria set to default thresholds.

### 3.4. Electrochemistry and ECL Measurement

Electrochemical characterization was conducted with a HYZ-3002 analyzer (HeYongzhong Electronic Technology, Xi’an, China) equipped with a photomultiplier tube (PMT) maintained at 700 V bias voltage. ECL spectral data acquisition utilized an electrogenerated chemiluminescence spectrum system (FORTEC Technology Co., Ltd., HongKong, China) featuring 121 L/nm dispersion grating coupled with a thermoelectrically cooled Andor CCD detector (DU401A-BR-DD, −80 °C operational temperature). The three-electrode system comprised a 3 mm glassy carbon working electrode (WE) paired with dual platinum elements functioning as counter (CE) and reference electrodes (REs). A ferrocene (Fc) redox couple served as the internal reference for potential calibration throughout electrochemical measurements.

Prior to experimentation, glassware underwent sequential purification: 4 hours immersion in alkaline solution (5% KOH/isopropanol) followed by equivalent acid treatment (5% HCl aqueous). The working electrode surface preparation involved sequential polishing with 0.3 → 0.2 → 0.05 μm alumina suspensions to achieve optical-grade finish, succeeded by ultrasonic cleaning in nitric acid (0.1 M) and deionized water, then argon drying. Electrode activation verification was performed via cyclic scanning in 1 mM K_3_[Fe(CN)_6_]/0.2 M potassium chloride until achieving <70 mV peak separation for the redox couple. Auxiliary electrodes received ultrasonic treatment in an acetone–isopropanol–water sequence (2 min each) followed by 120 °C desiccation and ambient cooling.

For ECL measurements, anhydrous acetonitrile solutions containing 0.05 mM target analyte and 0.1 M TBAP electrolyte were prepared under an inert atmosphere using glovebox techniques. Specialized Teflon cap sealing components ensured air-free conditions during electrochemical/spectral measurements.

## 4. Conclusions

This work demonstrates the successful design of a rigid oligo(phenylenevinylene)-bridged TADF dimer (BN-MOPV) that achieves high-performance ECL through synergistic molecular engineering and co-reactant optimization. The dimer exhibits analogous MR-TADF characteristics with narrowband blue–green emission (493 nm, FWHM = 22 nm) and a high photoluminescence quantum yield (PLQY = 67%), attributable to its spatially separated HOMO-LUMO distributions confirmed by DFT calculations. These short-range charge transfer (SRCT) characteristics ensure suppressed non-radiative decay and minimized singlet-triplet energy gaps (Δ*E*_ST_), aligning with recent advances in TADF systems where structural rigidity enhances spectral purity. Co-reactant-assisted ECL with TPrA achieves a remarkable 51% ECL efficiency, surpassing both annihilation-driven (11%) and BPO-mediated pathways (1%). This disparity arises from TPrA’s superior ability to stabilize radical intermediates and mediate efficient energy transfer, while BPO’s limited redox mediation capacity leads to parasitic recombination. Spectral analysis of BPO-enhanced ECL (λ_ECL_ = 500 nm) confirms exclusive singlet-excited-state emission, eliminating exciplex contributions—a critical feature of high-fidelity electroluminescence. These findings underscore the dual role of molecular rigidity in suppressing aggregation-induced quenching and co-reactant engineering in optimizing exciton harvesting. The work provides a blueprint for developing next-generation ECL materials, emphasizing the integration of TADF molecular design with electrochemical tailoring to overcome energy loss pathways.

## Figures and Tables

**Figure 1:**
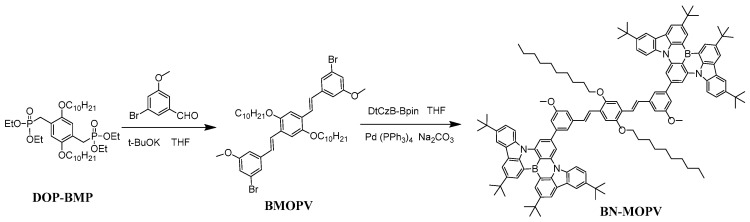
Synthetic routes to compound BN-MOPV.

**Table 1:** Summary of photophysical and electrochemical properties of BN-MOPV.

Compound	Abs ^a^ [nm]	λ_FL_^b^ [nm]	λ_PH_^c^ [nm]	FWHM ^d^ [nm]	Φ_PL_^e^ [%]	E_g_^f^ [eV]	E_S1_^g^ [eV]	E_T1_^h^ [eV]	ΔE_ST_ [eV]
BN-MOPV	328,400,472	493	517	22	67(88)	2.77	2.58	2.38	0.20

^a^ Peak wavelength of absorption peak in toluene solution (1 × 10^−5^ M). ^b^ Peak wavelength of fluorescence emission in toluene solution (1 × 10^−5^ M). ^c^ Peak wavelength of phosphorescence spectrum in toluene solution (1 × 10^−5^ M). ^d^ Full width at half maxima of fluorescence emission. ^e^ Absolute PLQYs evaluated under air (and argon). ^f^ Optical bandgaps were estimated from the onset wavelengths of the UV–vis using the formula E_S_ = 1240/λ_onset, absorption_. ^g^ Calculated from the intersection of normalized UV–vis absorption and fluorescence spectra using the formula E_S_ = 1240/λ _intersection_. ^h^ Calculated from the onset of phosphorescence spectra using the formula E_T_ = 1240/λ_onset, phosphorescence_.

## Data Availability

The data presented in this study are available from the corresponding author upon reasonable request.

## Supplementary Materials

The following supporting information can be downloaded at: https://www.mdpi.com/article/10.3390/molecules30081718/s1, Figures S1–S13: ^1^H and ^13^C NMR spectra; Figure S14: Mass spectrum of BN-MOPV; Figure S15: PL spectra of BN-MOPV under an air and argon; Table S1: HOMO-LUMO of BN-MOPV; Figure S16: CV and ECL-voltage curve of TBAP; Table S2: Coordinates of BN-MOPV at an optimized S0 geometry.
