# Supplementary material for: Synthesis and Electrochemiluminescence of a Di-Boron Thermally Activated Delayed Fluorescence Emitter
Source: Molecules. 2025 Apr 11;30(8):1718. doi: 10.3390/molecules30081718 (PMC12029476; doi:10.3390/molecules30081718)
Supplement: Supplementary file 1 [file molecules-30-01718-s001.zip › molecules-3557271-supplementary.pdf]

Supporting Information for

Synthesis and Electrochemiluminescence of a Di-Boron Thermally Activated Delayed Fluorescence Emitter

Xiaojie Zhou [1], Jun Cheng [2,*] and Hongbo Wang [1,*]

[1] Key Laboratory of Flexible Optoelectronic Materials and Technology (Ministry of Education), Center for International Cooperation and Disciplinary Innovation in Sustainable Chemical Engineering, School of Optoelectronic Materials and Technology, Jianghan University, Wuhan 430056, China; 17771987269@163.com
[2] Department of Chemistry, University of Liverpool, Crown Street, Liverpool L69 7ZD, UK
* Correspondence: juncheng@liverpool.ac.uk (J.C.); hongbo.wang@jhun.edu.cn (H.W.)

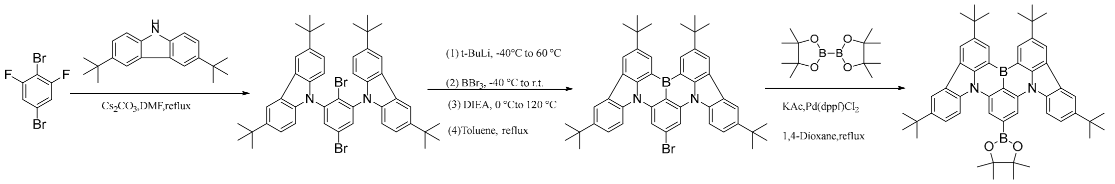

1 2 DtCzB-Bpin

Scheme S1. Synthetic route for compound DtCzB-Bpin.

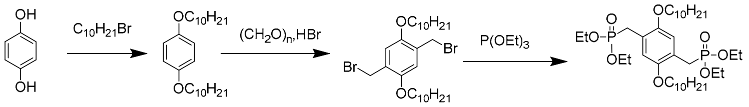

3 4 DOP-BMP

Scheme S2. Synthetic route for compound DOP-BMP.

Synthesis of compound 1: In a 500 mL three-necked round-bottomed flask equipped with a magnetic stir bar, sequentially add 3,6-di-tert-butylcarbazole (12.29 g, 44 mmol, 2.2 eq) and cesium carbonate (32.50 g, 100 mmol, 5 eq). Secure the flask on an iron stand. Purge the system with argon to remove air and create an inert atmosphere. Add 60 mL of DMF to dissolve the reagents completely, forming a suspension. Place the flask in a water bath and stir at 70 °C for 20 minutes. Once the mixture is homogeneous, slowly introduce

2,5-dibromo-1,3-difluorobenzene (5.40 g, 20 mmol, 1 eq) through the side neck of the flask. Gradually increase the temperature to 155 °C and maintain this temperature for 12 hours. After the reaction, cool the system to room temperature. Remove the inorganic salt precipitate by filtration using a Büchner funnel. Use a rotary evaporator to remove the high-boiling N,N-dimethylformamide under reduced pressure. Dissolve the residue in dichloromethane and purify via silica gel column chromatography (elution system: petroleum ether/dichloromethane = 5:1, v/v). Finally, obtain compound 1 as a white crystalline solid (13.61 g, 86% yield). ^{1}H NMR (400 MHz, Chloroform-*d*) δ 9.05 (s, 2H), 8.41 (s, 2H), 8.30 (s, 2H), 8.21 (d, J = 11.3 Hz, 4H), 7.64 (d, J = 8.7 Hz, 2H), 1.67 (s, 18H), 1.53 (s, 18H).

Synthesis of compound 2: In a 120 mL two-necked round-bottomed flask equipped with a magnetic stir bar and a spherical condenser, add compound 1 (3.95 g, 5 mmol, 1 eq) and super-dry mesitylene (50 mL), maintaining an argon atmosphere within the flask. With the flask in an ice bath, slowly add n-butyllithium (BuLi, 2.2 mL, 5.5 mmol, 1.1 eq) dropwise. Maintain the reaction system in a -78 °C acetone bath for 20 minutes, then remove the cooling bath and allow the system to react for 60 minutes as it warms to room temperature naturally. At -40 °C, accurately inject boron tribromide (BBr3, 0.95 mL, 10 mmol, 2.0 eq) via syringe and stir for 15 minutes. Subsequently, increase the temperature to room temperature at a rate of 5 °C/min and react for an additional 30 minutes. Finally, inject DIEA (1.6 mL, 10 mmol, 2 eq) in an ice bath, seal the flask and stir at 180 °C for 12 hours. After cooling to room temperature, quench the reaction by adding 5 mL of pre-cooled deionized water in an ice bath, then perform liquid–liquid extraction with dichloromethane. Combine the organic phases, dry over anhydrous sodium sulfate and concentrate under reduced pressure using a rotary evaporator to obtain a brown viscous residue. Purify the residue using a silica gel chromatography column packed with glass, eluting with a gradient of petroleum ether/dichloromethane (initial ratio 4:1, v/v). Collect the product fractions, concentrate under reduced pressure and obtain compound 2 as a yellow crystalline solid. ^{1}H NMR (400 MHz, Chloroform-*d*) δ 8.08 (s, 4H), 7.68 (s, 2H), 7.45 (d, J = 8.6 Hz, 4H), 7.07 (d, J = 8.5 Hz, 4H), 1.40 (s, 38H).

Synthesis of compound DtCzB-Bpin: In a 100 mL round-bottomed flask with a magnetic stirrer and a spherical condenser, compound 2 (500 mg, 0.696 mmol, 1 eq), 30 mL of 1,4-dioxane, pinacol boronic acid (441 mg, 1.74 mmol, 2.5 eq), potassium acetate (204 mg, 2.088 mmol, 3 eq) and dichlorobis (diphenylphosphinoferrocene)palladium (51 mg, 0.0696 mmol, 0.1 eq) were added in sequence under argon. The mixture was refluxed at 110 °C for 12 hours. After cooling to room temperature, it was extracted with dichloromethane, and the combined organic layers were concentrated under vacuum. The residue was purified by column chromatography using petroleum ether/dichloromethane (4:1, v/v) to yield DtCzB-Bpin as a yellow powder (479 mg, 90%). ^{1}H NMR (400 MHz, Chloroform-*d*) δ 9.13 (s, 2H), 8.78 (s, 2H), 8.53 (d, J = 8.7 Hz, 2H), 8.47 (s, 2H), 8.26 (s, 2H), 7.73 (d, J = 8.8 Hz, 2H), 1.67 (s, 18H), 1.54 (s, 18H), 1.49 (s, 12H).

Synthesis of compound 3: In a 100 mL round-bottomed flask, add 1,4-benzenediol (475 mg, 4.31 mmol, 1 eq), DMSO (30 mL), 1-bromodecane (2 g, 9.042 mmol, 2.1 eq) and KOH (1.83 g, 32.6 mmol, 7.56 eq) under argon. Reflux at 25 °C for 11 hours in a thermostated magnetic stirrer. After cooling, transfer the reaction mixture to a separatory funnel and perform three-phase extraction with dichloromethane (3 × 30 mL). Dry the combined organic phases with anhydrous sodium sulfate and concentrate to 1/5 of the original volume using rotary evaporation. Dissolve the brown residue in a minimal amount of dichloromethane and purify via silica gel column chromatography with a gradient elution (PE/DCM =1:1, v/v), monitoring by TLC. Collect the crude product, evaporate the solvent and recrystallize from diethyl ether/n-hexane (1:3) to obtain compound 3 as white prisms (874 mg, 52% yield). ^{1}H NMR (400 MHz, Chloroform-*d*) δ 6.82 (s, 4H), 3.89 (t, *J* = 6.5 Hz, 4H), 1.81 – 1.69 (m, 4H), 1.56 (s, 1H), 1.43 (d, *J* = 7.1 Hz, 4H), 1.29 (d, *J* = 13.4 Hz, 24H), 0.88 (t, *J* = 6.2 Hz, 5H). ^{13}C NMR (101 MHz, Chloroform-*d*) δ 153.20, 115.40, 68.68, 31.91, 29.59 (d, *J* = 2.9 Hz), 29.53 – 29.24 (m), 26.07, 22.69, 14.13.

Synthesis of compound 4: In a 100 mL round-bottomed flask with a magnetic stirrer at room temperature, add compound 3 (590 mg, 1.8 mmol), paraformaldehyde (165 mg, 5.49 mmol) and glacial acetic acid (40 mL). Dissolve 33% HBr (10 mL, 55 mmol) in glacial acetic acid (20 mL) and add this solution dropwise to the flask. Stir vigorously and reflux at 55 °C for 22 hours. After cooling to room temperature, quench the reaction by pouring the mixture into pre-cooled ice water (0-5 °C, 200 mL), resulting in a flocculent precipitate. Filter under vacuum using a sintered glass funnel, and wash the precipitate with ice methanol (10 mL) followed by diethyl ether (5 mL). Dry the product in a vacuum desiccator for 24 hours to obtain compound 4 as white crystals (750 mg, 81% yield based on compound 4). ^{1}H NMR (400 MHz, Chloroform-*d*) δ 6.83 (d, *J* = 11.4 Hz, 2H), 4.53 (s, 4H), 3.98 (t, *J* = 6.3 Hz, 4H), 1.85 – 1.76 (m, 4H), 1.48 (dd, *J* = 14.0, 7.0 Hz, 5H), 1.28 (s, 24H), 0.88 (t, *J* = 5.4 Hz, 5H). ^{13}C NMR (100 MHz, Chloroform-*d*) δ 150.67, 127.52, 114.66, 69.02, 31.91, 29.58 (d, *J* = 2.9 Hz), 29.35 (d, *J* = 2.6 Hz), 28.77, 26.09, 22.69, 14.12.

Synthesis of compound tetraethyl ((2,5-bis(decyloxy)-1,4-phenylene)bis -(methylene)) -bis(phosphonate)(**OPC**): In a 100 mL flask with a magnetic stirrer, combine compound 4 (1.66 g, 2.89 mmol, 1 eq) and triethyl phosphite (1.92 g, 11.56 mmol, 4 eq). Place the reaction system in an oil bath at 130 °C and stir for 24 hours. After cooling to 25 °C, hot-filter the mixture through a Büchner funnel to remove catalyst residues. Transfer the filtrate to a separatory funnel and extract with dichloromethane (3×30 mL). Dry the organic layer with anhydrous sodium sulfate, concentrate using rotary evaporation and obtain OPC as a white product (1.6 g, 80% yield). ^{1}H NMR (400 MHz, Chloroform-*d*) δ 6.91 (s, 2H), 3.91 (t, *J* = 6.3 Hz, 4H), 3.22 (d, *J* = 20.3 Hz, 4H), 1.72 (d, *J* = 12.8 Hz, 12H), 1.24 (dd, *J* = 14.7, 7.1 Hz, 40H), 0.88 (t, *J* = 6.1

Hz, 6H). ^{13}C NMR (101 MHz, Chloroform-*d*) δ 150.43, 119.27, 114.94, 69.03, 62.12, 31.90, 29.74 – 29.24 (m), 26.14, 22.68, 16.51 – 16.18 (m), 14.12.

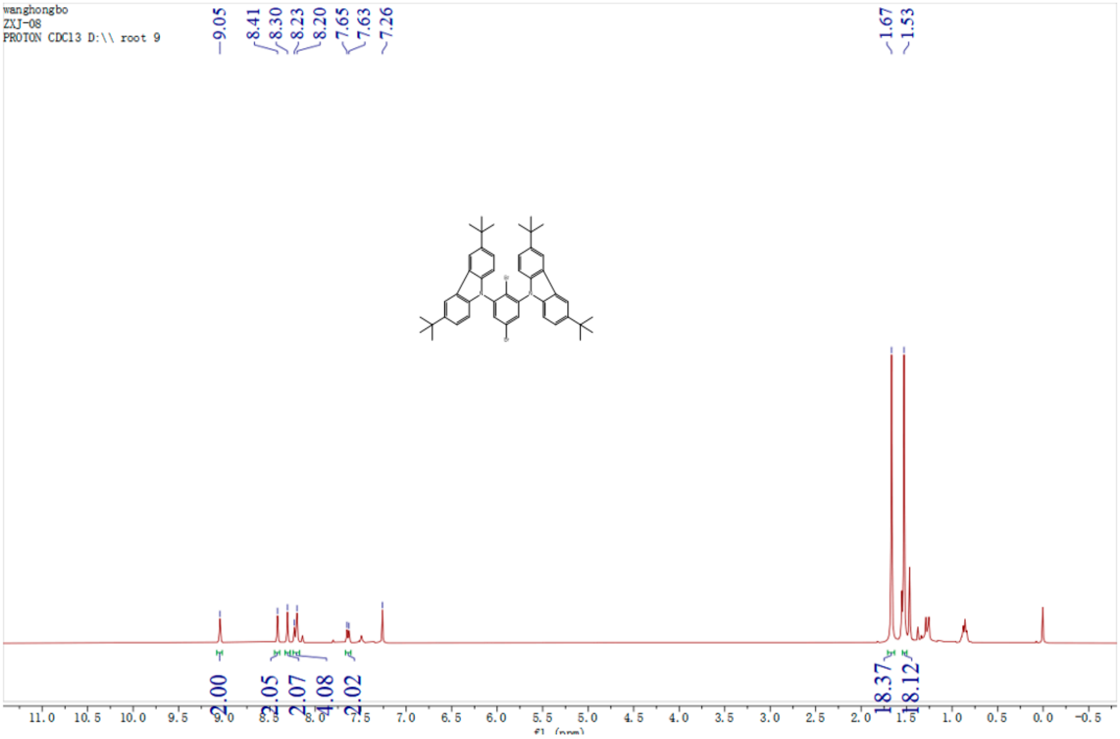

Figure S1. ^{1}H NMR (400 MHz, $CDCl_3$, 298 K) of 1.

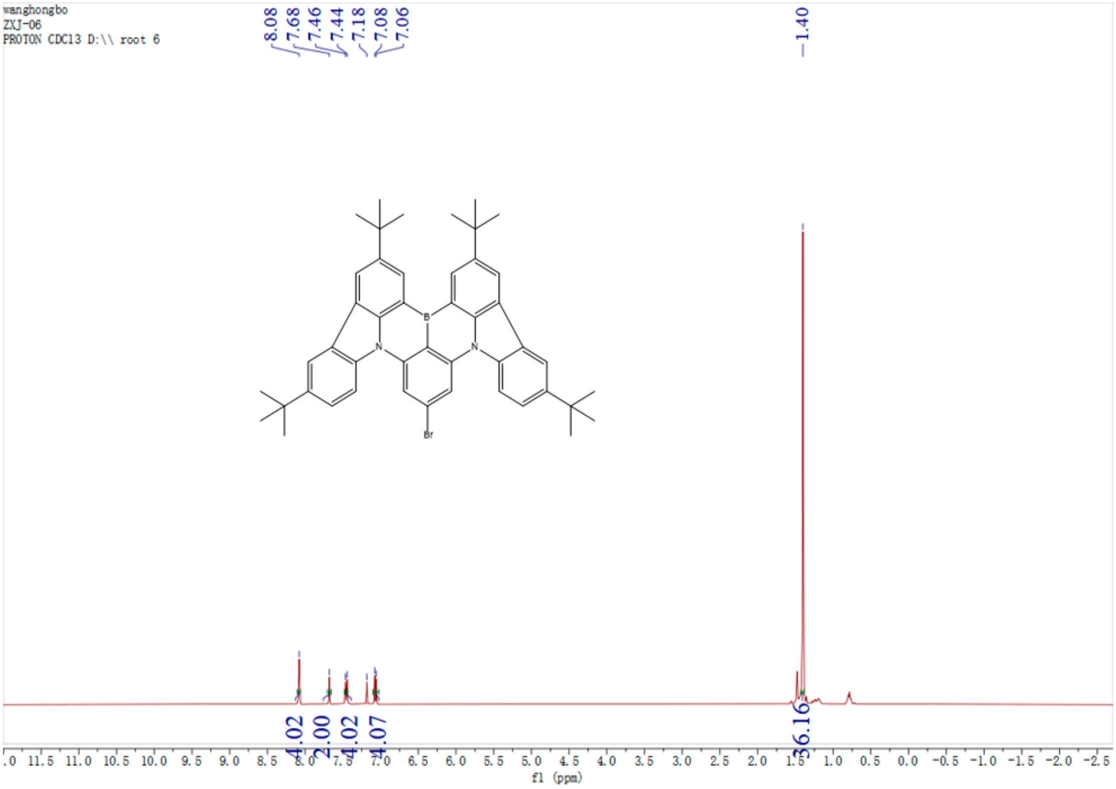

Figure S2. ^{1}H NMR (400 MHz, $CDCl_3$, 298 K) of 2.

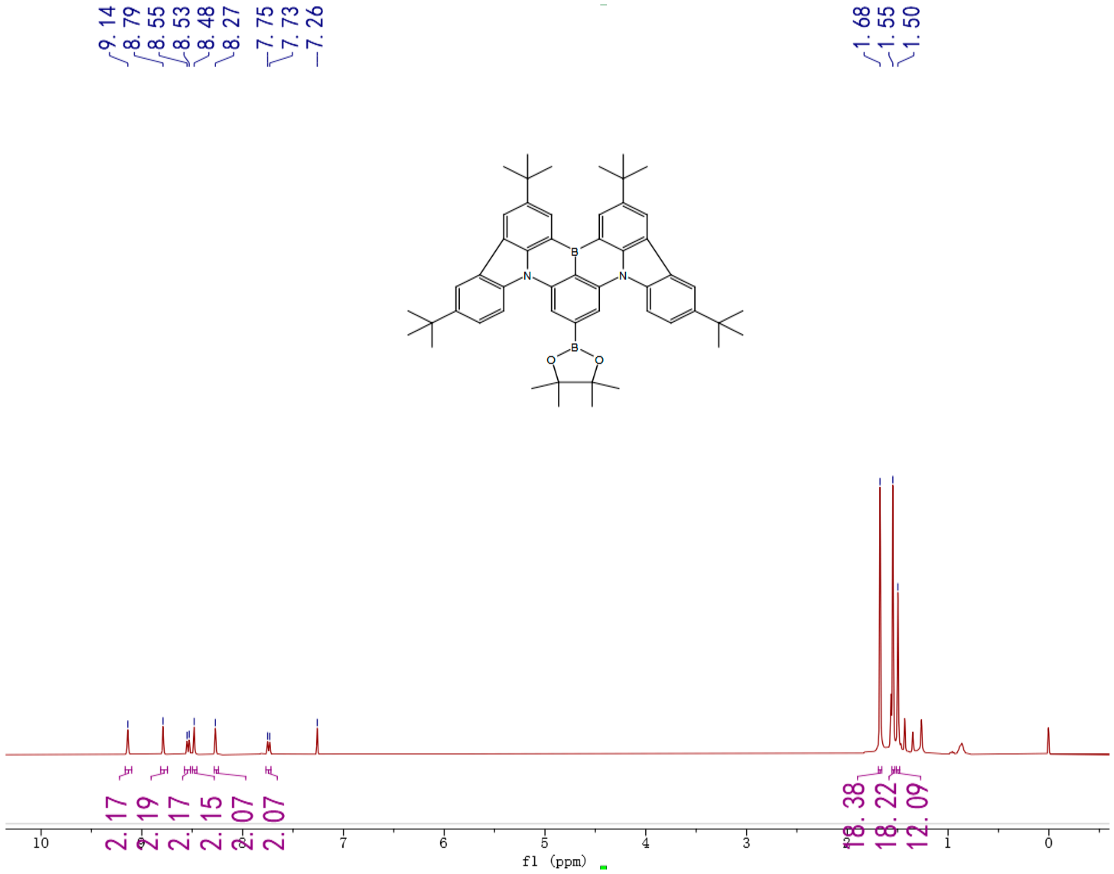

Figure S3. ^{1}H NMR (400 MHz, $CDCl_3$, 298 K) of DtCzB-Bpin.

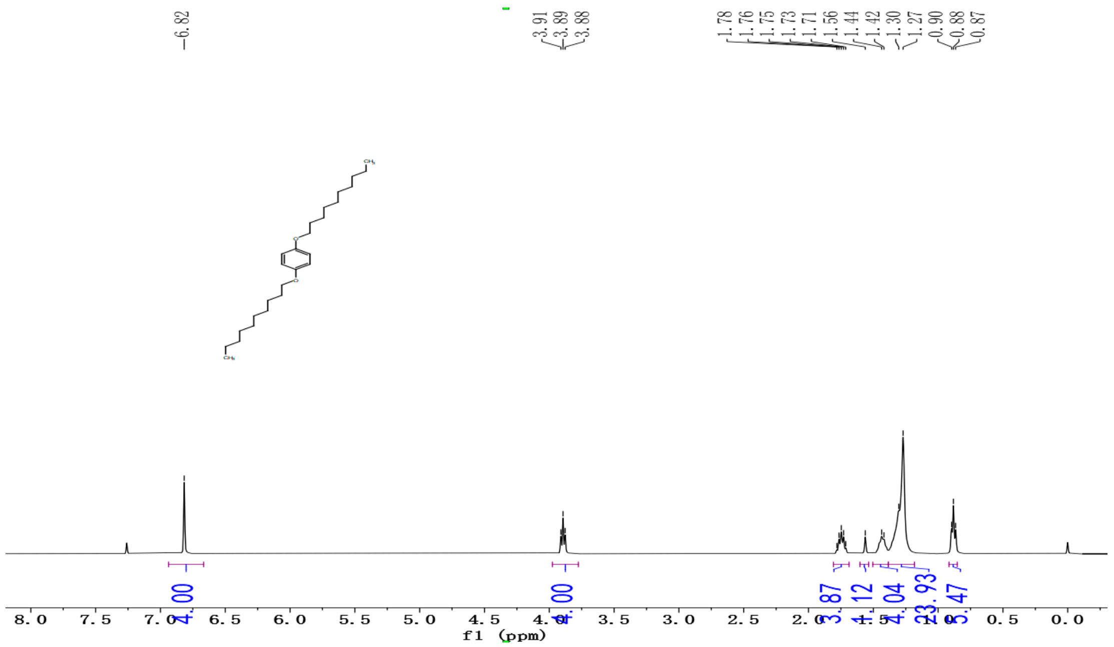

Figure S4. ^{1}H NMR (400 MHz, $CDCl_3$, 298 K) of 3.

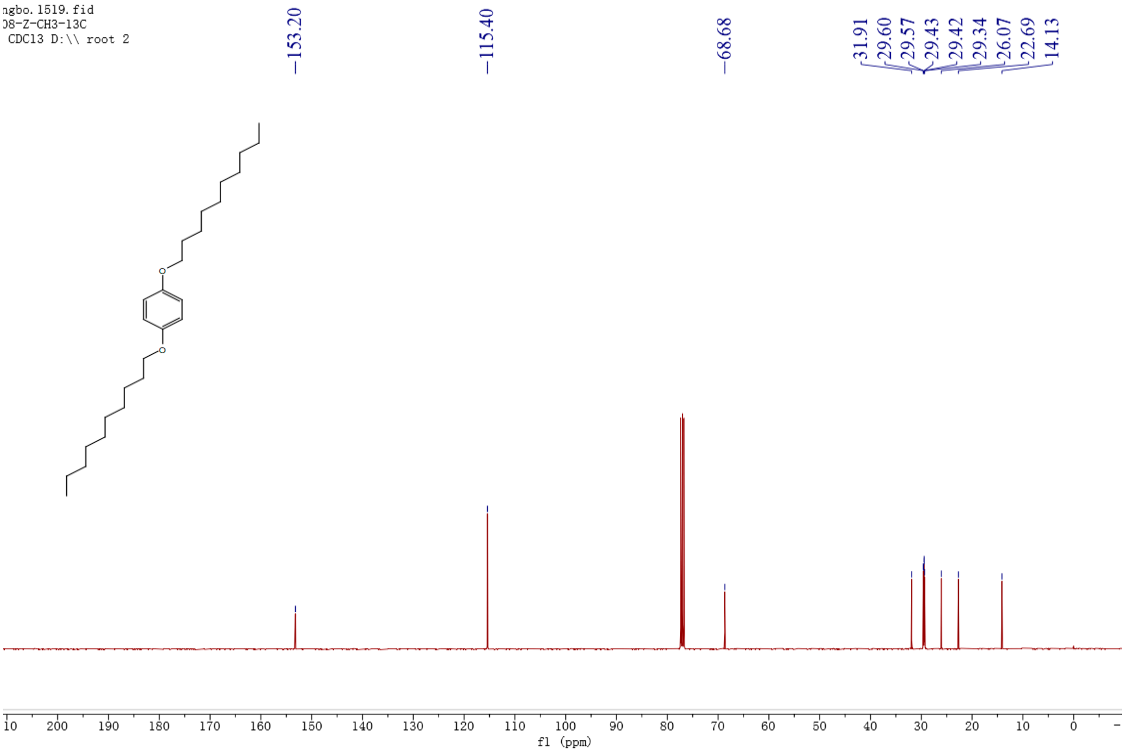

Figure S5. ^{13}C NMR (100 MHz, $CDCl_3$, 298 K) of 3.

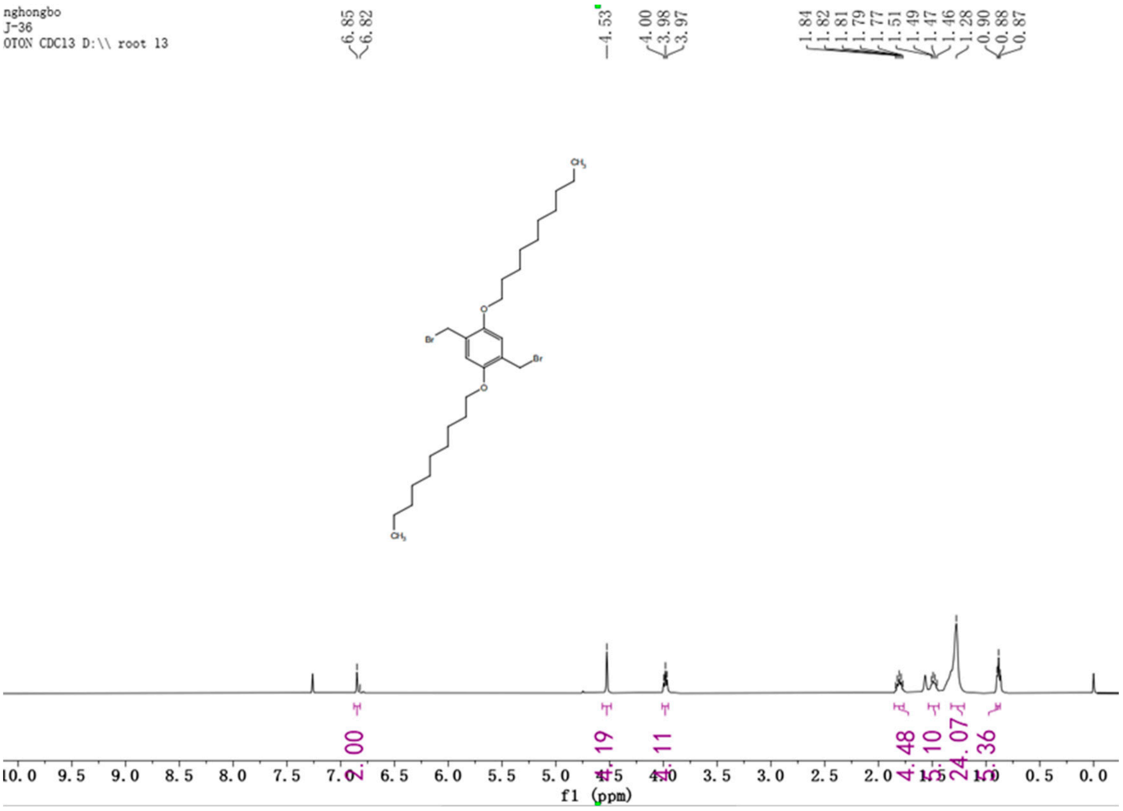

Figure S6. ^{1}H NMR (400 MHz, $CDCl_3$, 298 K) of 4.

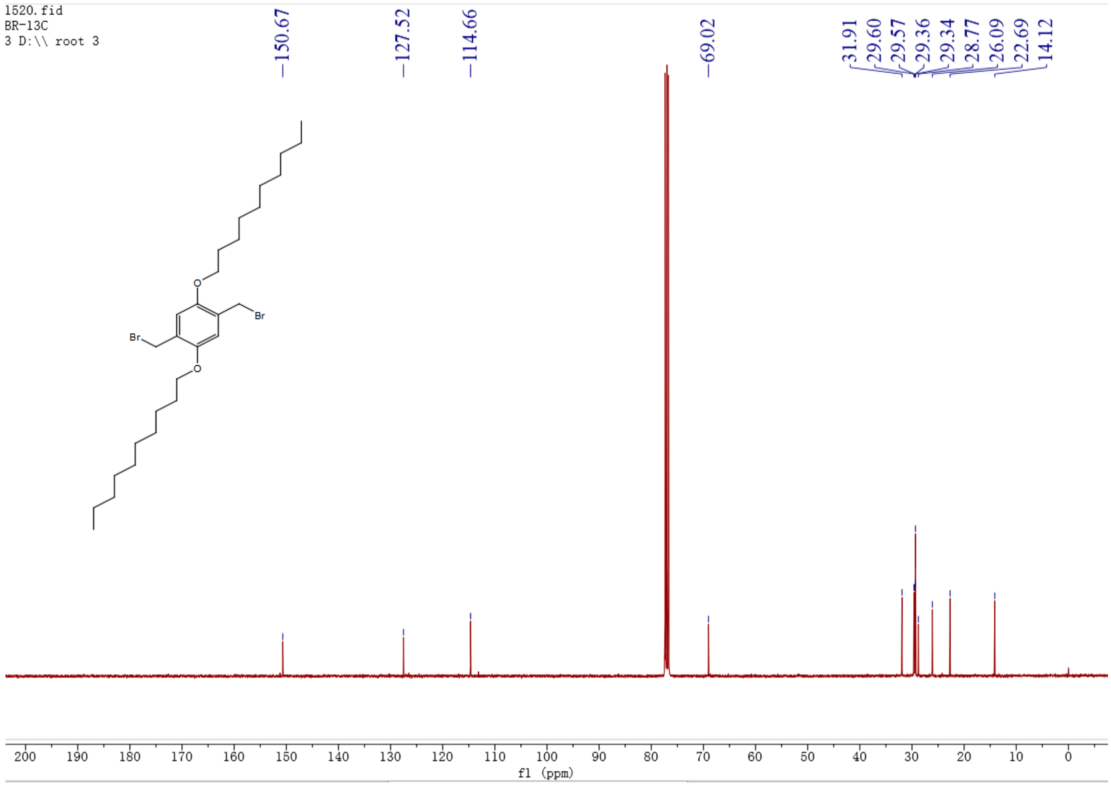

Figure S7. ^{13}C NMR (100 MHz, $CDCl_3$, 298 K) of 4.

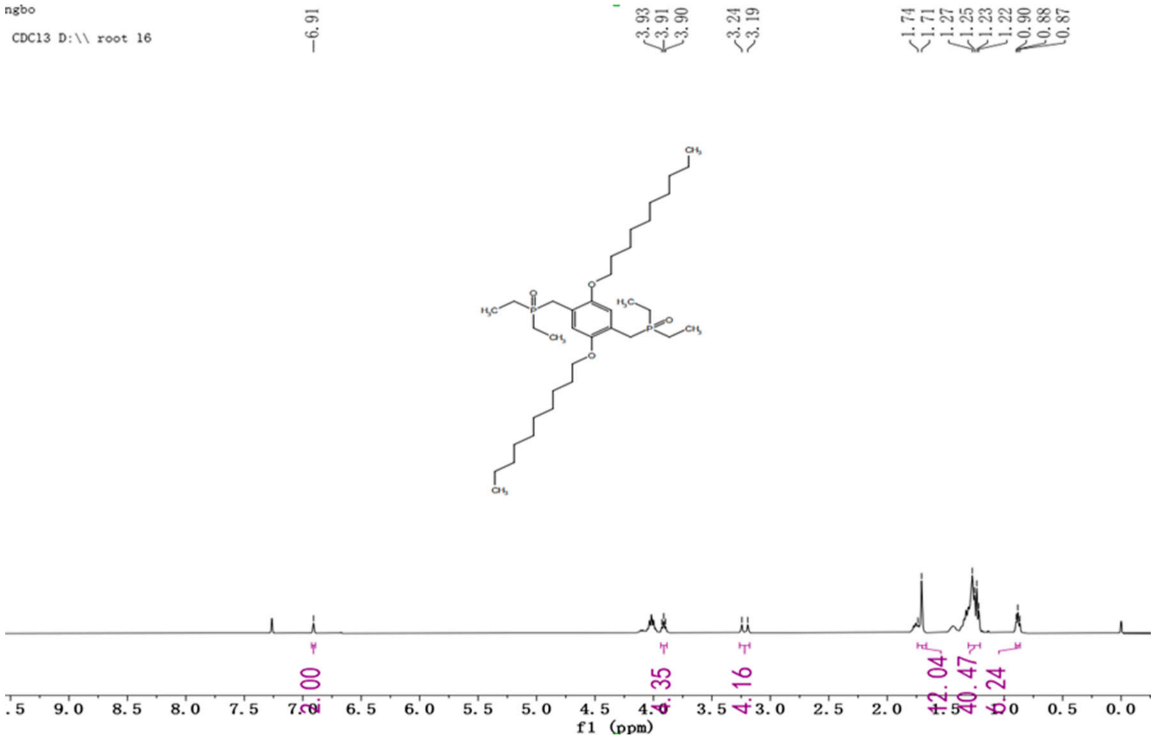

Figure S8. ^{1}H NMR (400 MHz, $CDCl_3$, 298 K) of DOP-BMP.

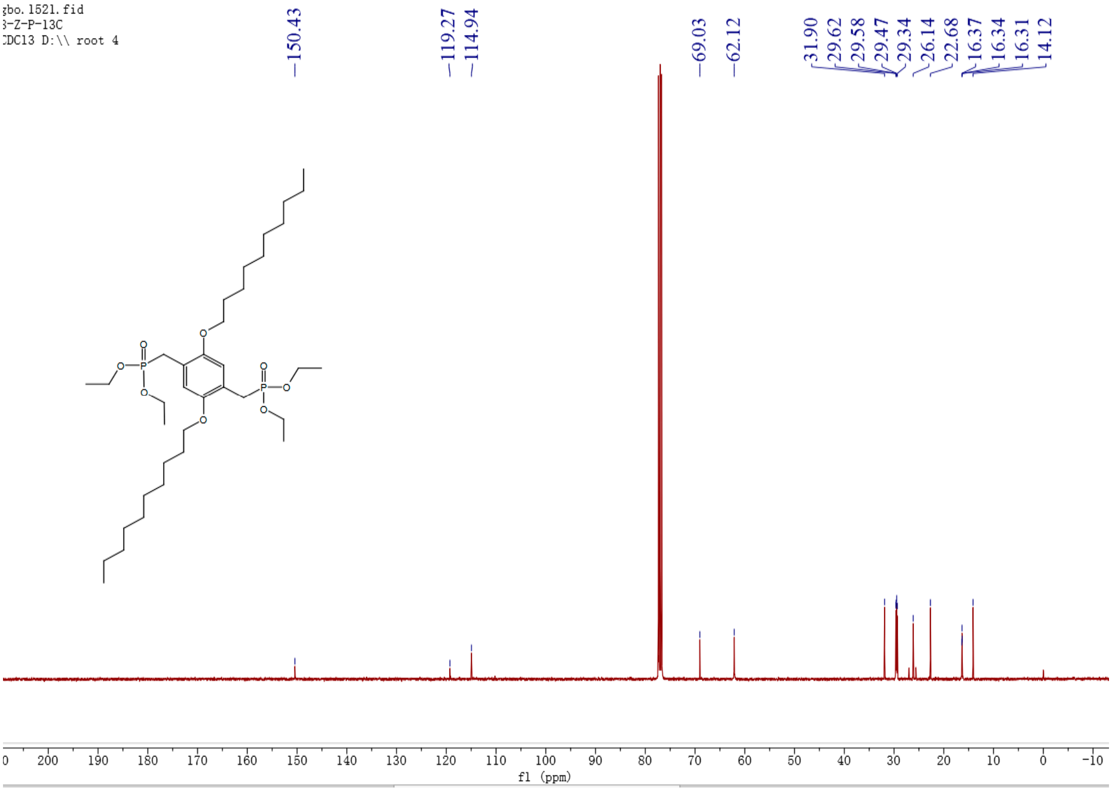

Figure S9. ^{13}C NMR (100 MHz, $CDCl_3$, 298 K) of DOP-BMP.

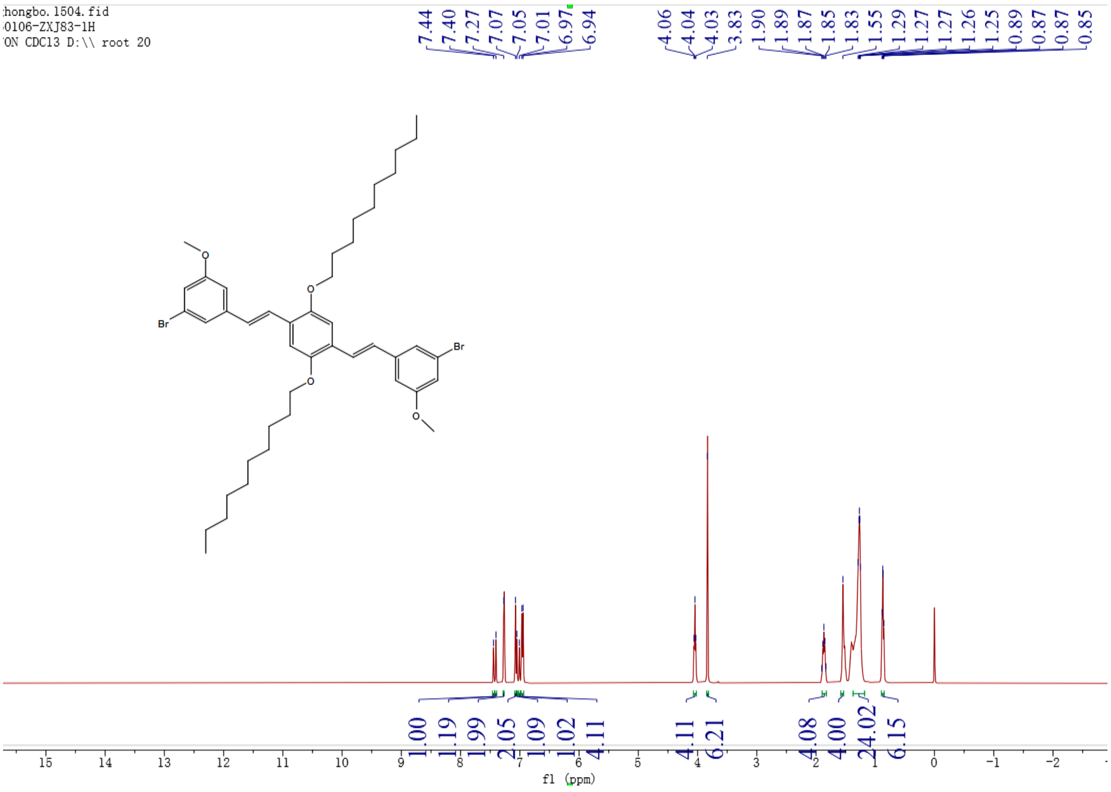

Figure S10. ^{1}H NMR (400 MHz, $CDCl_3$, 298 K) of BMOPV.

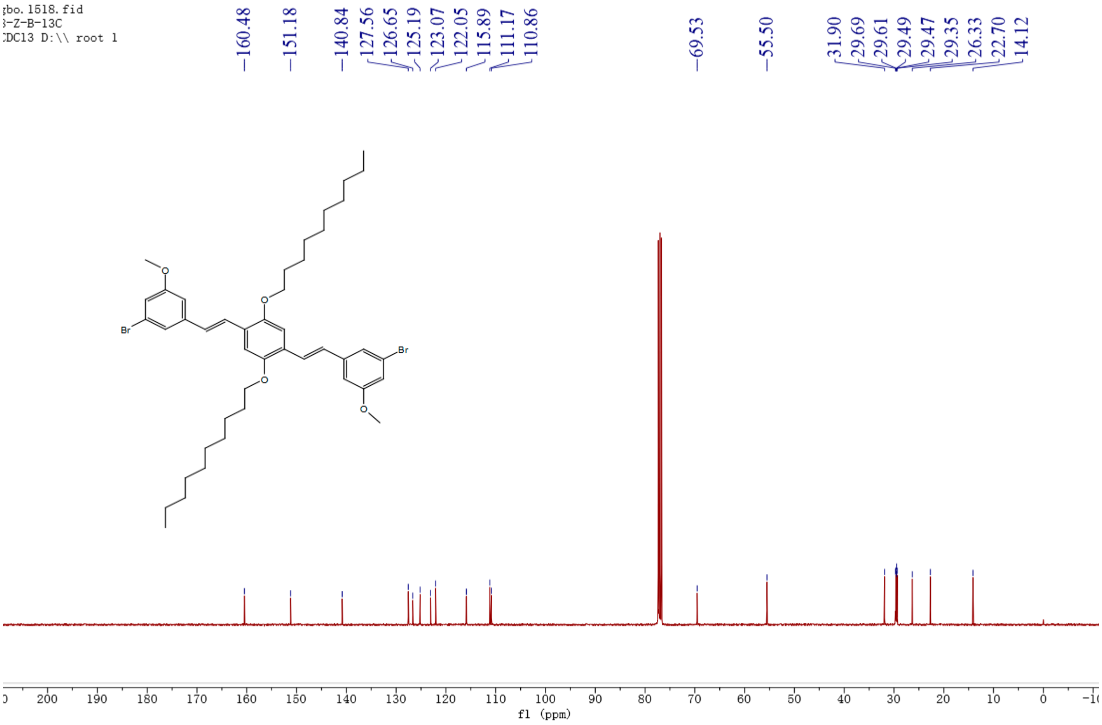

Figure S11. ^{13}C NMR (100 MHz, $CDCl_3$, 298 K) of BMOPV.

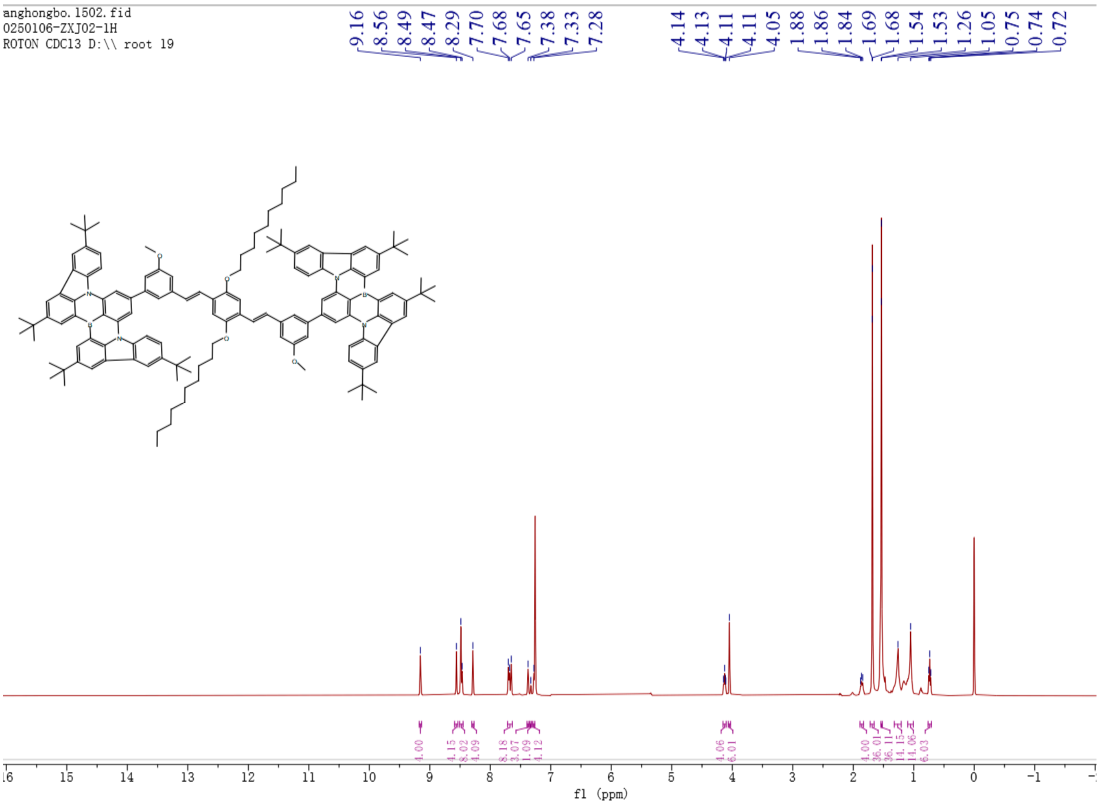

Figure S12. $^{1}H^{1}$NMR (400 MHz, $CDCl_3$, 298 K) of BN-MOPV.

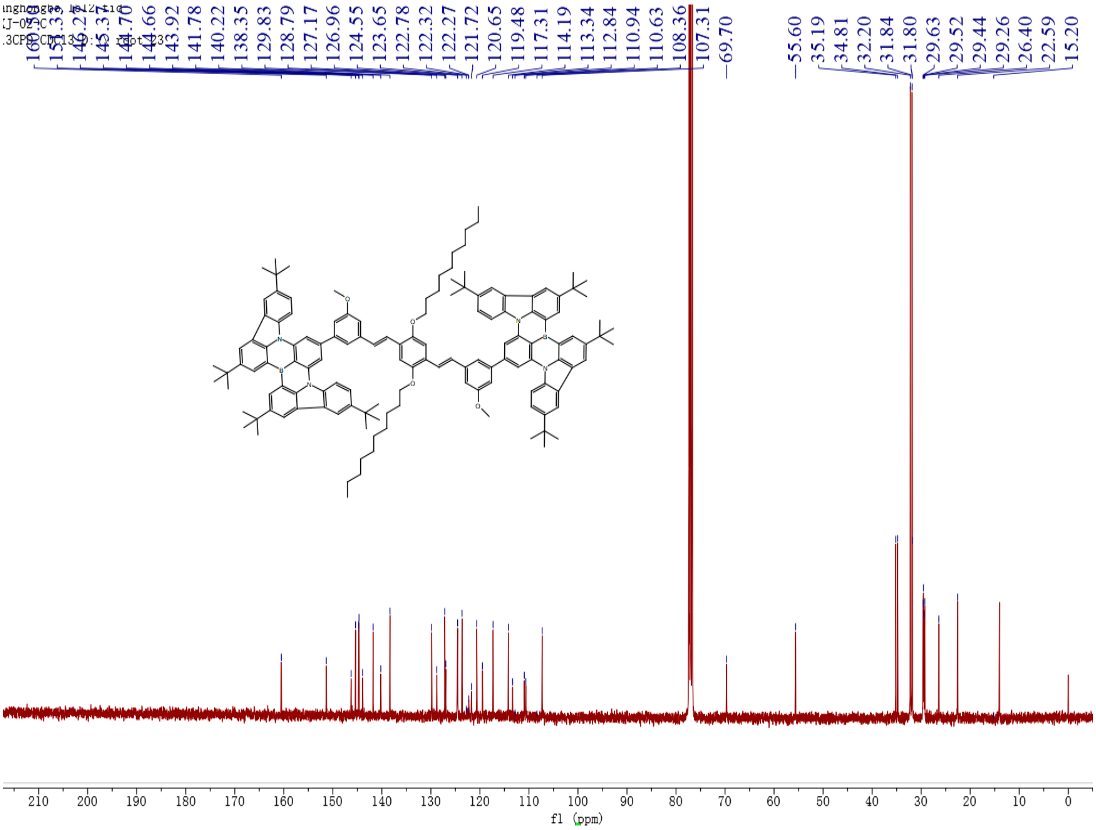

Figure S13. ^{13}C NMR (100 MHz, $CDCl_3$, 298 K) of BN-MOPV.

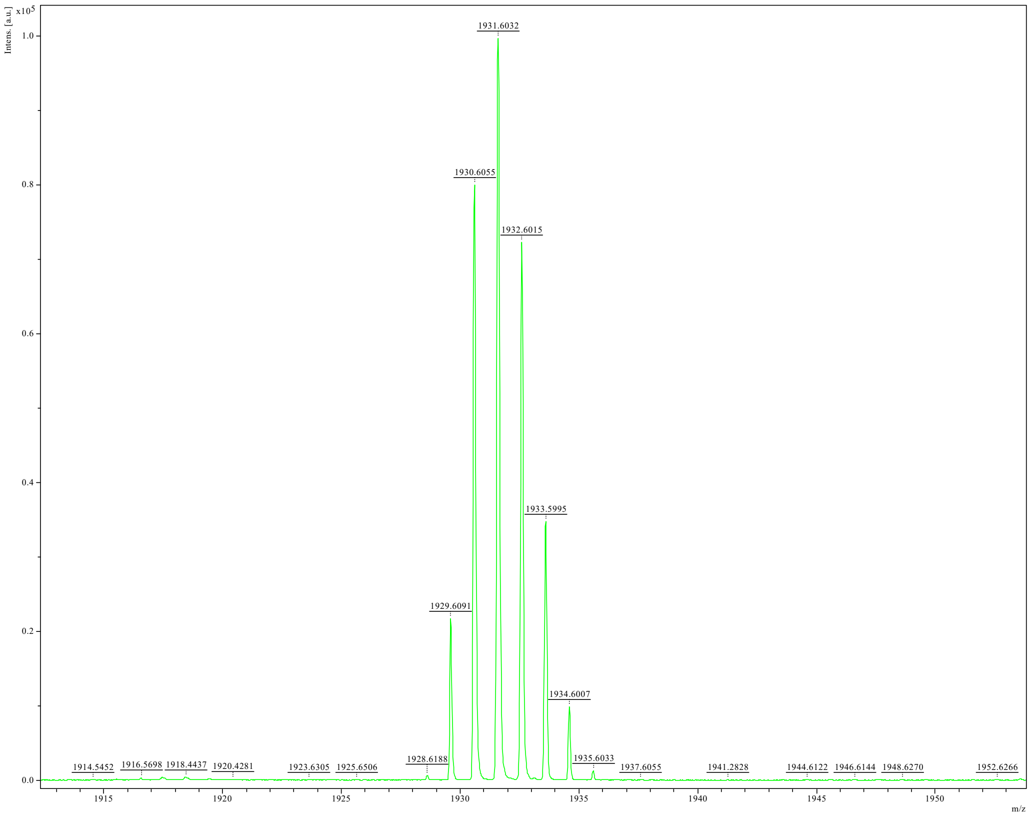

Figure S14. MALDI-TOF MS spectrum of BN-MOPV.

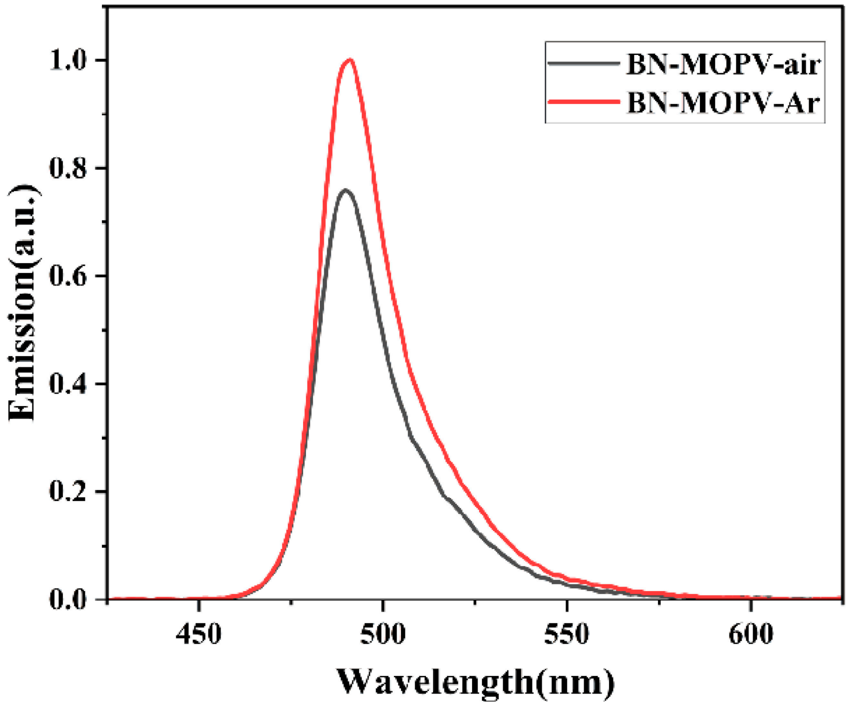

Figure S15. Emission spectra of BN-MOPV in toluene solutions (10^{-5} M) under an air and argon atmosphere.

Table S1. HOMO-LUMO of BN-MOPV.

Compound	HOMO/ LUMO [a] [eV]	HOMO/LUMO [b] [eV]	$HOMO_{-1}$ /$LUMO_{-1}$ [b] [eV]	E_{gap} [a] [eV]	$E^{b}_{HOMO/LUMO}$ [eV]	$E^{b}_{HOMO_{-1}/LUMO}$ [eV]	$E^{b}_{HOMO/LUMO_{-1}}$ [eV]
BN-MOPV	-5.2/-2.5	-5.25/-2.10	-5.29/-2.04	2.7/3.15	3.15	3.20	3.21

[a] The HOMO energy level was calculated from the measured oxidation potential (E_{ox}) using the formula $E_{HOMO} = -(4.8 + E_{ox})$; the LUMO energy level was derived from the reduction potential (E_{red}) using the formula $E_{LUMO} = -(4.8 + E_{red})$. [b] Theoretical DFT calculations provided additional values.

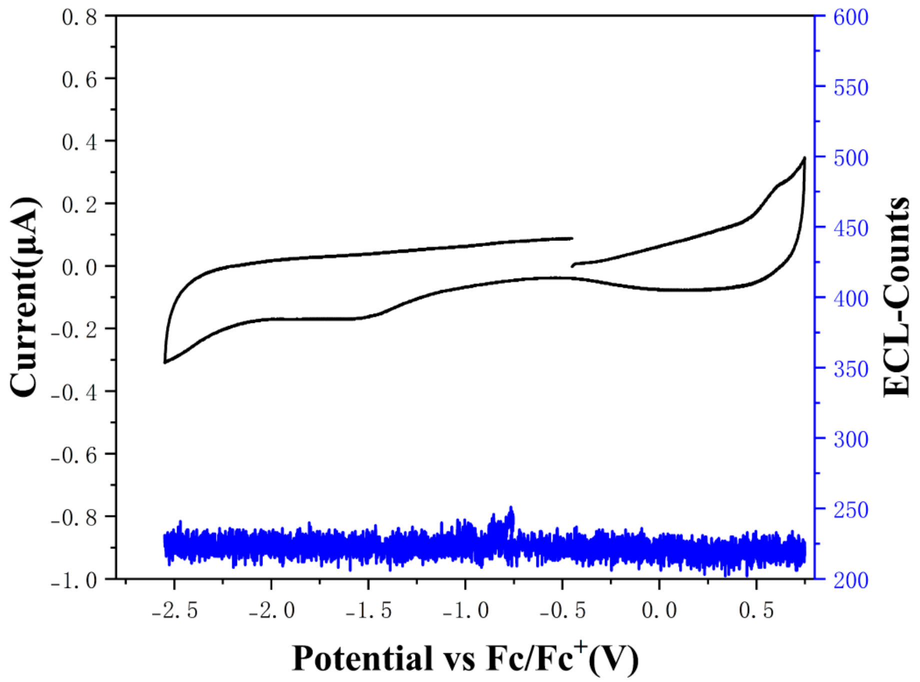

Figure S16. CV and ECL-voltage curve of 0.1M TBAP in acetonitrile and benzene (1:1) with 0.1M TBAP recorded at a scan rate of 0.1V/s positively.

Table S2. Cartesian coordinates of BN-MOPV at an optimized S0 geometry.

atom	x	y	z	atom	x	y	z
C	-7.58142	5.01827	-5.07712	H	-8.37783	3.17285	-3.51093
O	-6.31415	4.45024	-4.77275	H	-2.64373	1.86793	-3.08913
C	-6.26120	3.38359	-3.91814	H	-3.86411	-0.43081	-1.48468
C	-4.98108	2.86821	-3.67575	H	-2.60114	-2.17388	-0.90368
C	-4.79489	1.76702	-2.82893	H	1.61009	0.12250	-2.03128
C	-5.93074	1.19062	-2.22030	H	-9.31134	2.94660	-1.34048
C	-7.21063	1.69596	-2.45533	H	-7.71251	-0.91711	-2.15377
C	-7.37844	2.80455	-3.31109	H	-14.66470	-1.09709	0.95250
C	-3.42469	1.28112	-2.61762	H	-15.49801	3.06935	1.61889
C	-3.07122	0.17549	-1.92191	H	-14.10716	5.36818	1.46203
C	-1.73636	-0.37694	-1.68905	H	-10.09222	6.55399	0.45507
C	-1.66615	-1.67456	-1.13200	H	-9.50064	4.28800	-0.08790
C	-0.45667	-2.31846	-0.88164	H	-7.70344	-2.37428	-3.27197
C	0.77403	-1.67910	-1.18786	H	-6.71897	-4.43754	-4.01744
C	0.69826	-0.39052	-1.74064	H	-9.32917	-6.72250	-1.46559
C	-0.50809	0.26144	-1.97674	H	-11.36418	-6.22647	0.21104
C	-10.49390	1.29522	-0.60004	H	-13.47667	-2.73171	1.56135
C	-9.40275	1.87615	-1.25677	H	-17.47356	0.44803	-0.27526
C	-8.39887	1.07326	-1.80779	H	-16.85434	-1.10772	0.29760
C	-8.51336	-0.31961	-1.75115	H	-18.40425	-0.50143	0.90258
C	-9.65113	-0.91579	-1.19585	H	-17.11287	2.28859	3.01356

C	-10.66990	-0.12512	-0.57465	H	-17.67017	2.44258	1.33215
B	-11.90262	-0.77380	0.09015	H	-18.53863	1.39981	2.46475
N	-11.45935	2.09306	0.03840	H	-13.83859	7.05203	3.01508
N	-9.81831	-2.31195	-1.21865	H	-12.15935	7.36975	3.47727
C	-12.68081	1.56393	0.46007	H	-13.24558	8.71872	3.08332
C	-11.49950	3.49342	0.27546	H	-14.59294	7.45765	0.55801
C	-10.80114	-2.94167	-0.45286	H	-13.99023	9.11835	0.67957
C	-9.04963	-3.33172	-1.84151	H	-13.42316	8.05773	-0.62750
C	-13.02226	0.20484	0.50539	H	-10.50705	8.35363	1.77278
C	-14.33859	-0.06249	0.93695	H	-11.01751	8.62392	0.09180
C	-15.25563	0.93233	1.32797	H	-11.68428	9.61658	1.39428
C	-14.83013	2.27345	1.30698	H	-12.19012	-7.17570	2.11075
C	-13.54195	2.59442	0.87861	H	-13.90924	-7.49531	2.36938
C	-12.79061	3.83042	0.76591	H	-13.29323	-7.31120	0.72295
C	-13.10872	5.14802	1.09321	H	-15.26093	-4.15876	1.45080
C	-12.15702	6.17109	0.96943	H	-15.06589	-5.49625	0.30754
C	-10.87179	5.80411	0.53046	H	-15.65374	-5.80927	1.95436
C	-10.52564	4.49084	0.18962	H	-5.54899	-6.94554	-1.92363
C	-8.04473	-3.29292	-2.81576	H	-5.22818	-7.90168	-3.38584
C	-7.49470	-4.49643	-3.25913	H	-5.23119	-6.13283	-3.46515
C	-7.90783	-5.75956	-2.78391	H	-8.50133	-7.16075	-5.10941
C	-8.94312	-5.78192	-1.84401	H	-6.99552	-6.26847	-5.37451
C	-9.51671	-4.59112	-1.38540	H	-6.96084	-8.03378	-5.24869
C	-10.63348	-4.33780	-0.49601	H	-7.69003	-8.33227	-1.60138
C	-11.50024	-5.15051	0.23561	H	-8.88985	-8.42958	-2.90919
C	-12.53389	-4.57679	0.99833	H	-7.30789	-9.19535	-3.09687
C	-12.68029	-3.17594	0.97360	H	1.93940	-3.43500	-0.74526
C	-11.84015	-2.30876	0.24420	H	3.35793	-0.73201	-1.05927
C	-16.67801	0.52684	1.76749	H	5.62090	-0.64044	-0.54791
C	-17.39123	-0.20323	0.60309	H	7.99703	-4.21006	-0.11462
C	-17.54074	1.74108	2.16507	H	3.78511	-4.51434	-0.88723
C	-12.53451	7.62089	1.33032	H	5.42006	-7.73337	-0.61623
C	-12.97142	7.69063	2.81408	H	4.25744	-6.58338	0.10486
C	-13.70566	8.08773	0.43161	H	4.55593	-6.57130	-1.66348
C	-11.36202	8.60181	1.13263	H	9.37439	-2.62829	-1.41856
C	-13.50228	-5.42892	1.84505	H	7.18499	-0.16872	1.28252
C	-13.19770	-6.93719	1.74947	H	14.25170	2.33190	-0.06083
C	-14.95549	-5.20664	1.35939	H	15.85965	-0.73317	-2.61447
C	-7.23661	-7.03757	-3.32134	H	15.05913	-3.15585	-3.46402
C	-5.72136	-6.99797	-3.00542	H	11.64591	-5.72101	-2.90368
C	-7.43554	-7.12619	-4.85434	H	10.54759	-4.05807	-1.55642
C	-7.82143	-8.31625	-2.69001	H	6.21236	1.38492	1.49150
C	2.03828	-2.37250	-0.94596	H	4.72778	2.76587	2.78578
C	3.26789	-1.80964	-0.92787	H	8.04995	4.90705	4.49489

C	4.54552	-2.49802	-0.71284	H	10.72408	4.91941	4.23719
C	5.69854	-1.72390	-0.51287	H	13.61277	2.76097	1.89238
C	6.95542	-2.31768	-0.30245	H	16.73897	1.10448	-3.68257
C	7.05075	-3.71168	-0.29987	H	15.83679	2.61243	-3.46811
C	5.90724	-4.50012	-0.50496	H	17.60013	2.59103	-3.25544
C	4.66303	-3.90530	-0.71113	H	17.80741	-0.05772	-1.60926
O	6.12220	-5.85162	-0.48423	H	18.64342	1.45518	-1.22808
C	5.01525	-6.72161	-0.67757	H	17.61815	0.67164	-0.00733
C	10.52362	-1.05368	-0.47972	H	15.67676	3.71396	-1.14898
C	9.37001	-1.81157	-0.71472	H	16.41603	2.92445	0.26142
C	8.16502	-1.47734	-0.08890	H	17.43612	3.59440	-1.01715
C	8.09743	-0.35165	0.73848	H	12.16925	-6.34951	-5.07476
C	9.21644	0.47326	0.90516	H	13.03258	-5.12957	-6.02356
C	10.47732	0.13374	0.31851	H	13.60766	-6.80999	-5.99914
B	11.73103	1.00923	0.53550	H	14.88597	-6.77504	-2.44909
N	11.75573	-1.41955	-1.04921	H	13.28002	-7.33745	-2.93767
N	9.14182	1.64395	1.68088	H	14.69638	-7.77299	-3.90626
C	12.84771	-0.54992	-1.04972	H	16.17263	-5.09396	-3.90468
C	12.13261	-2.57375	-1.78792	H	15.87261	-6.11846	-5.31365
C	10.28960	2.33825	2.06829	H	15.41281	-4.41161	-5.35973
C	8.03239	2.28088	2.29992	H	13.16532	6.46040	2.35858
C	12.94500	0.65787	-0.35015	H	12.19222	6.63348	3.82730
C	14.15042	1.37234	-0.55163	H	13.93711	6.92406	3.88977
C	15.20169	0.91431	-1.36185	H	14.91846	4.59364	2.17319
C	15.05751	-0.33828	-1.99636	H	15.57124	5.08391	3.74088
C	13.88867	-1.07586	-1.84196	H	15.09259	3.40170	3.48004
C	13.43459	-2.37104	-2.31167	H	13.60563	3.56352	5.57273
C	14.06451	-3.35556	-3.07965	H	14.19476	5.22727	5.77262
C	13.43165	-4.57709	-3.33203	H	12.45563	4.89730	5.75283
C	12.15690	-4.77319	-2.75975	H	6.51328	6.55218	4.86896
C	11.50312	-3.80676	-1.99412	H	5.16493	6.41081	6.00349
C	6.64994	2.10552	2.16737	H	6.58678	5.37869	6.20217
C	5.79567	2.92509	2.90651	H	5.03337	3.32872	6.19752
C	6.25295	3.93587	3.77825	H	3.66542	4.45238	6.05151
C	7.63587	4.12571	3.86640	H	3.84105	3.11249	4.90714
C	8.51476	3.32161	3.13375	H	4.86955	6.19021	2.92593
C	9.95736	3.35448	2.98720	H	3.73745	4.82906	2.95371
C	10.97281	4.14149	3.51995	H	3.57267	6.13369	4.13844
C	12.31533	3.93026	3.13929	H	-16.09937	0.07328	3.83458
C	12.58611	2.93123	2.19043	H	-17.60431	-0.71343	3.31319
C	11.59101	2.11022	1.60782	H	-16.04113	-1.33460	2.76297
C	16.50004	1.71900	-1.57747	H	-13.64104	-3.95835	3.48250
C	16.67744	2.02125	-3.08584	H	-14.07888	-5.61001	3.94738
C	17.71170	0.89501	-1.07678	H	-12.37389	-5.17293	3.70870

C	16.49674	3.06310	-0.82313	H	-2.16452	-4.43130	-0.90431
C	14.07318	-5.69413	-4.17653	H	-2.14981	-3.68792	0.71618
C	13.16368	-6.01354	-5.38818	H	-0.54805	-6.16889	-0.11058
C	14.24059	-6.96910	-3.31404	H	-2.08771	-6.20336	0.74579
C	15.46187	-5.29708	-4.71470	H	-1.06399	-4.95269	2.66687
C	13.42030	4.80162	3.77130	H	0.47697	-4.89909	1.82057
C	13.15938	6.29202	3.44208	H	0.57909	-7.41380	1.84535
C	14.82719	4.44145	3.25508	H	-0.96576	-7.47073	2.68424
C	13.41484	4.61033	5.30798	H	0.03492	-6.21270	4.61819
C	5.23871	4.78386	4.56867	H	1.57843	-6.14498	3.77874
C	5.92509	5.83873	5.45853	H	1.69595	-8.65967	3.79181
C	4.39519	3.86184	5.48283	H	0.15211	-8.72743	4.63197
C	4.29976	5.52507	3.58569	H	1.14901	-7.46409	6.56651
C	-16.59737	-0.41891	2.99066	H	2.69205	-7.39280	5.72569
C	-13.39171	-5.01430	3.33276	H	2.81566	-9.90718	5.73900
O	-0.36557	-3.57066	-0.33629	H	1.27258	-9.97857	6.58048
C	-1.56022	-4.28081	0.00148	H	2.26873	-8.71365	8.51305
C	-1.16549	-5.61919	0.61336	H	3.81056	-8.64176	7.67185
C	-0.42956	-5.50303	1.95528	H	3.81132	-10.50265	9.35088
C	-0.05563	-6.86431	2.55759	H	3.95973	-11.16729	7.71548
C	0.67237	-6.75678	3.90473	H	2.40630	-11.23990	8.56280
C	1.05841	-8.11553	4.50553	H	0.81060	2.37552	-1.15695
C	1.78713	-8.00647	5.85219	H	-0.92093	2.65286	-0.86190
C	2.17713	-9.36407	6.45274	H	-1.03705	4.06571	-2.93096
C	2.90651	-9.25508	7.79899	H	0.67342	3.77039	-3.23533
C	3.29294	-10.61581	8.39101	H	1.25318	4.90904	-1.05850
O	-0.48001	1.52079	-2.54892	H	-0.45595	5.19492	-0.75094
C	-0.15054	2.59262	-1.64484	H	-0.59541	6.59782	-2.83339
C	-0.07181	3.88976	-2.43736	H	1.11299	6.31253	-3.14058
C	0.28848	5.09350	-1.55493	H	1.69231	7.43498	-0.96366
C	0.36869	6.41264	-2.33629	H	-0.01614	7.71821	-0.65479
C	0.72775	7.62072	-1.46027	H	-0.15806	9.12643	-2.73659
C	0.80594	8.94153	-2.23850	H	1.55088	8.84479	-3.04298
C	1.16229	10.15039	-1.36214	H	2.12609	9.96516	-0.86366
C	1.24117	11.47169	-2.13918	H	0.41703	10.24705	-0.55774
C	1.59622	12.68120	-1.26326	H	0.27781	11.65761	-2.63859
C	1.67301	13.99642	-2.04799	H	1.98719	11.37641	-2.94311
H	-7.37904	5.83979	-5.76653	H	2.55905	12.49645	-0.76462
H	-8.24045	4.28732	-5.56361	H	0.85062	12.77764	-0.46035
H	-8.07109	5.41026	-4.17595	H	1.92816	14.83919	-1.39408
H	-4.13346	3.33739	-4.16739	H	0.71413	14.22720	-2.53004
H	-5.81446	0.36287	-1.52861	H	2.43513	13.94389	-2.83630
